# A Family of Ethyl *N*-Salicylideneglycinate Dyes Stabilized by Intramolecular Hydrogen Bonding: Photophysical Properties and Computational Study

**DOI:** 10.3390/molecules26113112

**Published:** 2021-05-23

**Authors:** Larisa E. Alkhimova, Maria G. Babashkina, Damir A. Safin

**Affiliations:** 1 Institute of Chemistry, University of Tyumen, Volodarskogo Str. 6, 625003 Tyumen, Russia; 2 Institute of Condensed Matter and Nanosciences, Université Catholique de Louvain, Place L. Pasteur 1, 1348 Louvain-la-Neuve, Belgium; maria.babashkina@gmail.com; 3 Kurgan State University, Sovetskaya Str. 63/4, 640020 Kurgan, Russia; 4 Innovation Center for Chemical and Pharmaceutical Technologies, Ural Federal University Named after the First President of Russia B.N. Eltsin, Mira Str. 19, 620002 Ekaterinburg, Russia

**Keywords:** Schiff base, *N*-salicylidene aniline derivative, photophysical properties, solvatochromism, Hirshfeld surface analysis, DFT

## Abstract

In this work we report solvatochromic and luminescent properties of ethyl *N*-salicylideneglycinate (**1**), ethyl *N*-(5-methoxysalicylidene)glycinate (**2**), ethyl *N*-(5-bromosalicylidene)glycinate (**3**), and ethyl *N*-(5-nitrosalicylidene)glycinate (**4**) dyes. **1**–**4** correspond to a class of *N*-salicylidene aniline derivatives, whose photophysical properties are dictated by the intramolecular proton transfer between the OH-function and the imine N-atom, affording tautomerization between the enol-imine and keto-enamine forms. Photophysical properties of **1**–**4** were studied in different pure non-polar and (a)protic polar solvents as well as upon gradual addition of NEt_3_, NaOH, and CH_3_SO_3_H. The DFT calculations were performed to verify the structures of **1**–**4** as well as their electronic and optical properties.

## 1. Introduction

Schiff base is a condensation product of aldehyde and primary amine. When salicylaldehyde derivative is used as an aldehyde and amine is a monoamine derivative, the condensation yields a NO-Schiff base compound that may produce complexes with a great variety of metal ions upon deprotonation. Thus, Schiff bases are still of great importance in coordination chemistry, although more than a century has passed since their discovery [1,2,3]. Schiff bases have been known to be the most actively used ligands thanks to their ease of synthesis, availability, structural versatility, and solubility in common solvents. Moreover, Schiff bases are not only efficient ligands to coordinate/chelate many elements but can stabilize elements in various and specific oxidation states. It is also noteworthy that Schiff bases, as well as their corresponding complexes, can possess pronounced bioactivity [4,5,6], including against coronavirus [7]. The latter is becoming even more important since these days humanity is in dire need of drugs for COVID-19 treatment.

**Scheme 1:**
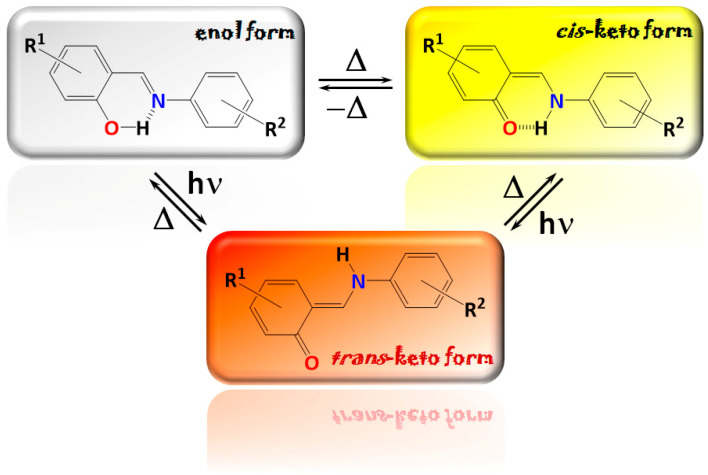
Isomeric forms and the corresponding color palette of *N*-salicylidene aniline derivatives.

Schiff bases fabricated from salicylaldehyde derivatives (*N*-salicylidene aniline derivatives) have been the main focus of our extensive studies due to their chromic properties and a broad color palette [8,9,10,11,12,13,14,15,16,17,18]. Particularly, these compounds are known to a possible intramolecular transfer of the OH proton to the imine N-atom affording tautomerization between the enol-imine and keto-enamine forms [19,20,21,22,23,24,25,26,27,28]. This phenomenon is usually responsible for the fascinating optical properties of these compounds. Notably, the enol-imine and keto-enamine forms can adopt either the *trans*- or *cis*-isomers, yielding a rich color palette from colorless to red through yellow (Scheme 1) [29,30]. These colors can be called by different external stimuli (light irradiation, temperature, solvent, pH, etc.). Thus, chromic properties are one of the main features of *N*-salicylidene aniline derivatives and are important for many practical applications [31,32,33,34,35].

Notably, due to the abovementioned tautomerization, *N*-salicylidene aniline derivatives can also possess the so-called ESIPT (excited state intramolecular proton transfer) [36], which can afford an equilibrium between the excited keto-enamine* and enol-imine* forms [29,30,37,38]. The keto-enamine form usually, emits at a lower energy than the enol-imine tautomer leading to dual fluorescence, which can be observed as two (or more) emission bands [29,30,37,38]. The ratio of these bands can be influenced by the nature of a solvent, which allows tuning the resulting emission color.

**Chart 1:**
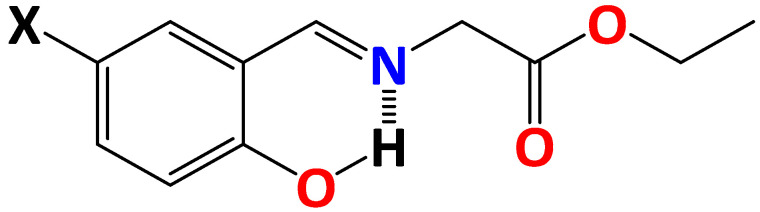
Diagrams of the discussed dyes **1** (X = H), **2** (X = OMe), **3** (X = Br) and **4** (X = NO_2_).

It should be noticed that we have recently initiated comprehensive studies of closely related *N*-salicylidene aniline derivatives **1**–**4**, derived from the ethyl ester of glycine (Chart 1) [17,18]. We found that **1**–**4** in the solid state each exhibits an enol-imine form. Notably, the electron-withdrawing NO_2_ substituent in **4** is mainly responsible for the formation of the *cis*-keto form isomer in EtOH, while dyes **1**–**3** adopt the enol-imine form in the same solvent. Additionally, only **4** is emissive under ambient conditions in the solid state, while **2** is emissive in EtOH.

Intrigued by the obtained results, herein, we continue our comprehensive studies of the dyes **1**–**4**. Particularly, to probe solvatochromism, we focused on the optical properties of these compounds in different solvents, as well as shedding more light on their crystal structures using the Hirshfeld surface analysis to examine in-depth the non-covalent interactions responsible for crystal packing, which might be responsible for the observed photophysical properties of **1**–**4** in the solid state.

## 2. Results and Discussion

Dyes **1**–**4** were produced according to the described synthetic procedure [18]. We first applied a Hirshfeld surface analysis [39] to study the intermolecular interactions in the reported crystal structures of **1**–**4** [17]. As a result, a set of 2D fingerprint plots [40] were generated using CrystalExplorer 3.1 [41]. In order to estimate the propensity of two chemical species being in contact, we also calculated the enrichment ratios (*E*) [42] of the intermolecular contacts.

The intermolecular H∙∙∙H, H∙∙∙C and H∙∙∙O contacts occupy a majority of a molecular surface of **1** (Table 1). There is a barely observed splitting of the shortest H∙∙∙H fingerprint at *d*_e_ + *d*_i_ ≈ 2.4 Å, which is due to the shortest contact being between three atoms, rather than for direct two-atom contact (Figure S1 in Supplementary Materials) [40]. The H∙∙∙C and H∙∙∙O shortest contacts are shown at *d*_e_ + *d*_i_ ≈ 2.7 and 2.3 Å, respectively (Figure S1). The latter contacts in the corresponding fingerprint plot are shown as two spikes and mainly correspond to the contacts formed by the carbonyl oxygen atom. The molecular surface of **1** is also characterized by a negligible proportion of the H∙∙∙N, C∙∙∙O and O∙∙∙O contacts, ranging from 0.6% to 2.4% (Table 1).

Substitution of the 5-hydrogen atom by the MeO function in **2** decreases a proportion of the H∙∙∙H and H∙∙∙C contacts by 2.8 and 1.7%, respectively, with the simultaneous increase of a proportion of the H∙∙∙O contacts up to 30.1% (Table 1). The H∙∙∙H and H∙∙∙C shortest contacts are now shown at *d*_e_ + *d*_i_ ≈ 2.2 and 2.9 Å, respectively (Figure S2). The H∙∙∙O contacts in the corresponding 2D fingerprint plot are also shown as two spikes with the shortest contacts at *d*_e_ + *d*_i_ ≈ 2.3 Å, also being formed by the carbonyl oxygen atom (Figure S2). The Hirshfeld molecular surface of **2** is further described by a very minor proportion of the H∙∙∙N, C∙∙∙C, C∙∙∙O and O∙∙∙O contacts, varying from 0.3 to 2.4% (Table 1).

In **3,** incorporation of the bromine instead of the hydrogen in **1** or the methoxy group in **2** significantly decreases a proportion of the H⋯H and H⋯C intermolecular contacts down to 35.4 and 13.7%, respectively. A proportion of the H⋯O contact remains almost the same as in **1** (Table 1). The H∙∙∙H, H∙∙∙C and H∙∙∙O shortest contacts are shown in the corresponding 2D fingerprint plots at *d*_e_ + *d*_i_ ≈ 2.2, 2.8, and 2.3 Å, respectively (Figure S3). The molecular surface of **3** is additionally characterized by a remarkable proportion of the H∙∙∙Br intermolecular contacts of 17.4%, with the shortest contacts at *d*_e_ + *d*_i_ ≈ 3.0 Å (Figure S3). Notably, the molecular surface of **3** is further described by a distinct proportion of the C∙∙∙C contacts of 4.1%, which is due to intermolecular π⋯π stacking interactions between the phenylene rings [17]. These contacts are shown on the 2D plot as a typical area at *d*_e_ = *d*_i_ ≈ 1.7–2.0 Å (Figure S3). Insignificant contributions from H···N, C···N, C···O, C···Br, N···O, O···O, and Br···Br contacts in **3** have also been revealed (Table 1).

Dye **4** contains the NO_2_ group in the same position as the corresponding substituent in **2** and **3** (Chart 1). The molecular surface of **4** is characterized by a dominant contribution from the H···O contacts of 44.1% with the shortest contacts, shown in the corresponding 2D fingerprint plot at *d*_e_ + *d*_i_ ≈ 2.3 Å mainly formed by both the NO_2_ and carbonyl oxygen atoms (Figure S4). The H∙∙∙H and H∙∙∙C contacts occupy 30.0 and 11.5%, respectively. Notably, the structure of **4**, similar to **3**, also exhibits a distinct proportion of the C∙∙∙C contacts of 4.9% due to the formation of intermolecular π⋯π stacking interactions between the phenylene rings [17]. These contacts are also shown on the 2D plot as a typical area at *d*_e_ = *d*_i_ ≈ 1.7–2.0 Å (Figure S4). Remaining contacts, namely H···N, C···N, C···O, N···O and O···O contacts, occupy a negligible proportion of the molecular surface of **4** (Table 1).

All the H∙∙∙X (X = C, N, O) contacts are highly favored in **1** and **2** since the corresponding enrichment ratios *E*_HX_ are significantly higher than unity (Table 1). This is explained by a relatively higher proportion of these contacts on the total Hirshfeld surface area over a corresponding proportion of random contacts *R*_HX_ (Table 1). However, only H⋯N and H⋯O intermolecular contacts are favored in **3** and **4**, while the H⋯C contacts on the surface of molecules are much less favored, which is related to a remarkably high enrichment of C⋯C contacts in **3** and **4** (Table 1), formed in aromatic stacking. Notably, both **3** and **4** are additionally characterized by highly favored H⋯Br and N⋯O contacts, respectively, and **3** is also described by less favored Br⋯Br contacts (Table 1). The H⋯H intermolecular contacts on the molecular surfaces of all compounds are less favored since the corresponding enrichment ratios *E*_HH_ are less than unity ranging from 0.85 to 0.90 (Table 1). The remaining contacts are significantly impoverished with the corresponding enrichment ratios less than 0.50 (Table 1).

We have also examined the optical properties of **1**–**4** in different solvents. We first probed such solvents as cyclohexane (non-polar), THF (polar aprotic), CH_3_CN (polar aprotic), and EtOH (polar protic) to study the enol-imine and keto-enamine tautomerization in the applied solvents. It is known that the dipole moments for the enol-imine isomers are smaller than those for the keto-enamine derivatives [43,44], which means that the keto-enamine tautomer is more prevalent in polar solvents.

The absorption spectra of **1–3** each contain bands corresponding to n→π* and π→π* transitions only in the UV region, regardless of the solvent (Figures S5–S7). Interestingly, the longest wavelength band in the spectra of **2** is remarkably red-shifted (Figures S5–S7). This is due to the electron-donating properties of the MeO fragment in comparison to the H and Br substituents in **1** and **3**, respectively. Furthermore, the spectra of **1**–**3** in EtOH exhibit an additional low-intense band, centered at about 400–420 nm (Figures S5–S7), which corresponds to the traces of the *cis*-keto-enamine form.

**Figure 1:**
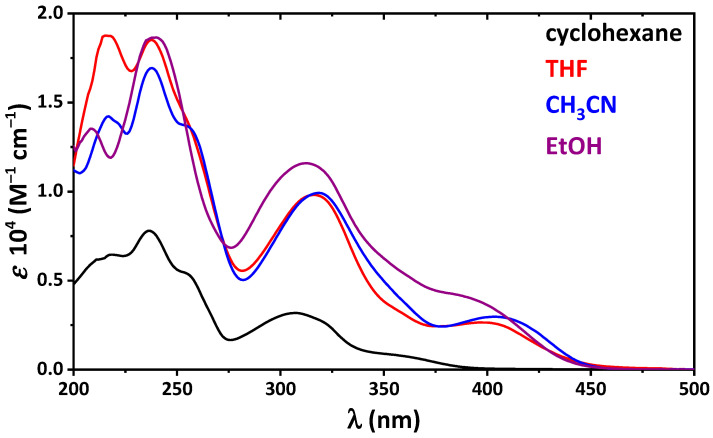
UV-vis spectra of **4** in the applied solvents.

The absorption spectra of **4** in the same solvents, except cyclohexane, contain an intense band in the visible region, which arises from the *cis*-keto-enamine form (Figure 1). Notably, in **4**, the electron-donating OH is involved in a through-resonance effect with the electron-withdrawing NO_2_ fragment. This leads to nitro–*aci*-nitro tautomerization, yielding an additional quinoid form [45].

**Figure 2:**
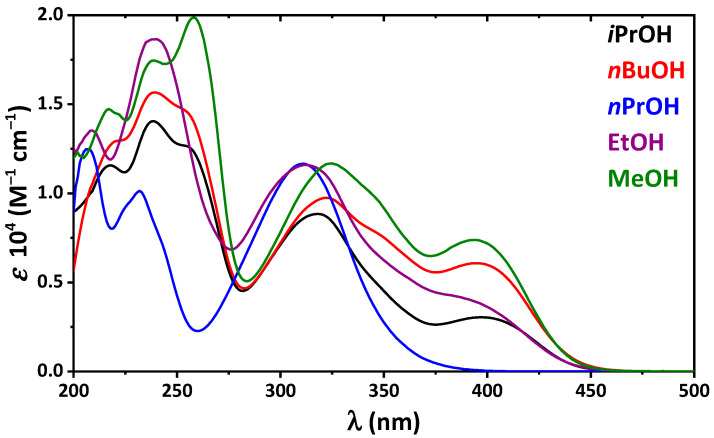
UV-vis spectra of **4** in the applied solvents.

Optical properties of **4** were also studied in a series of alcohols, namely MeOH, EtOH, *n*PrOH, *i*PrOH, and *n*BuOH. Interestingly, in the absorption spectra of **4** in all alcohols, except *n*PrOH, the band of the *cis*-keto-enamine form was observed with the most remarkable one in MeOH (Figure 2), which is the most polar and acidic within the applied alcohols. The most striking observation is the absence of the band of the *cis*-keto-enamine form in the spectrum of **4** in *n*PrOH (Figure 2). This might be explained by possible specific solute–solvent interactions.

**Figure 3:**
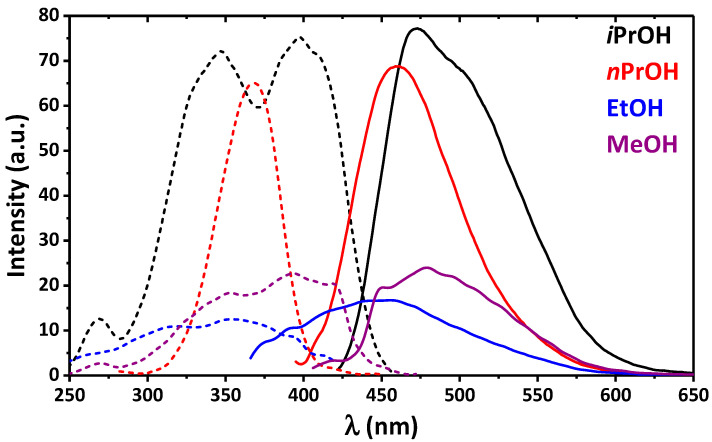
Emission (solid line) and excitation (dashed line) spectra of **4** in MeOH (λ_exc_ = 395 nm, λ_em_ = 480 nm), EtOH (λ_exc_ = 350 nm, λ_em_ = 440 nm), *n*PrOH (λ_exc_ = 360 nm, λ_em_ = 460 nm) and *i*PrOH (λ_exc_ = 400 nm, λ_em_ = 475 nm).

Recently, we established that **2** was emissive in EtOH [17]. The resulting emission of **2** in EtOH was due to two emission bands arising from two conformers of the *cis*-keto-enamine* form. Herein we report the emission properties of **4** in different alcohols. Interestingly, **4** was found to be emissive in *i*PrOH and *n*PrOH, while almost no emission was found in EtOH and MeOH (Figure 3).

The emission spectra of **4** in *i*PrOH and *n*PrOH exhibit an intense band at 475 and 460 nm, respectively. Notably, the former band is accompanied with an intense low-energy shoulder (Figure 3). The deconvolution process of the spectrum in *i*PrOH has allowed revealing three single bands at 463, 493, and 526 nm, and a ratio of these bands is 26.4, 30.0, and 43.6%, respectively (Figure S8). The same deconvolution of the emission spectrum of **4** in *n*PrOH also revealed three bands, but with the maxima remarkably shifted to higher energies (444, 466, and 496 nm) and with a ratio of 17.2, 46.2, and 36.6%, respectively (Figure S8). Based on the absorption and excitation spectra of **4** in *i*PrOH we were able to conclude that these three emission bands corresponded to two *cis*-keto-enamine* and *trans*-keto-enamine* conformers [8,9,10,11,12,13,14,15,16,17,18]. Contrarily, all three bands in the emission spectrum of **4** in *n*PrOH arose exclusively from the emission of different *cis*-keto-enamine* conformers.

**Figure 4:**
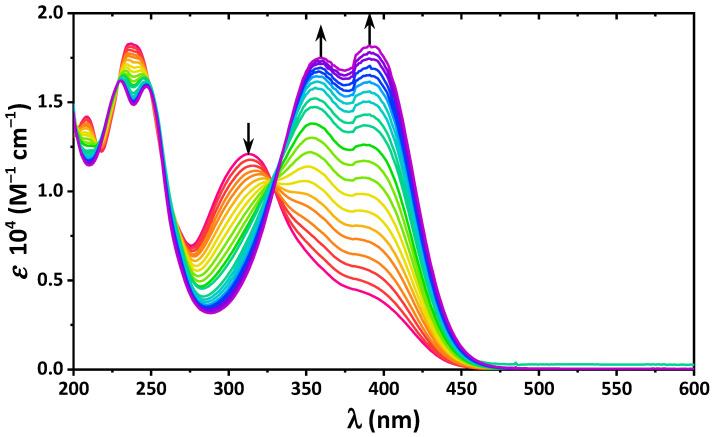
UV-vis spectra of **4** in EtOH upon gradual addition of NaOH up to 2 eqv., with a step of 0.1 eqv.

We next studied the solution of **4** upon the gradual addition of two different bases, namely NEt_3_ and NaOH, to probe the influence of the nature of bases (electronic properties and steric demands). Upon addition of both bases, the absorption band at 313 nm disappeared and two new bands, centered at about 360 and 390 nm, appeared (Figure 4 and Figure S9). Notably, the ratio of intensities of the new bands was in favor of the low-energy band when NaOH was used.

**Figure 5:**
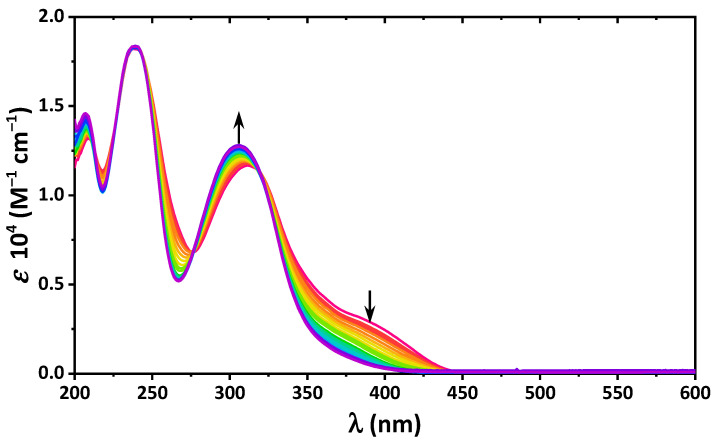
UV-vis spectra of **4** in EtOH upon gradual addition of CH_3_SO_3_H up to 2 eqv., with a step of 0.1 eqv.

The gradual addition of the methanesulfonic acid into a solution of **4** in EtOH decreased the intensity of the band of the *cis*-keto-enamine form, which was due to protonation of the imine N-atom, while the NO_2_ group remained unchanged, as seen from the high-energy band, which remained constant during the titration process (Figure 5). Protonation of the imine N-atom prevented the formation of the intramolecular O–H∙∙∙N bonding.

Since compound **3** also tends to clearly exhibit the *cis*-keto-enamine form in EtOH (Figure S7), although less pronounced than **4,** but more pronounced that **1** and **2**, its optical properties were further studied upon gradual addition of NaOH and CH_3_SO_3_H. In general, the behavior of a solution of **3** in EtOH in the presence of NaOH and CH_3_SO_3_H was similar to a solution of **4** but much less pronounced; however, emission spectra of **3** differed significantly from those of **4**.

**Figure 6:**
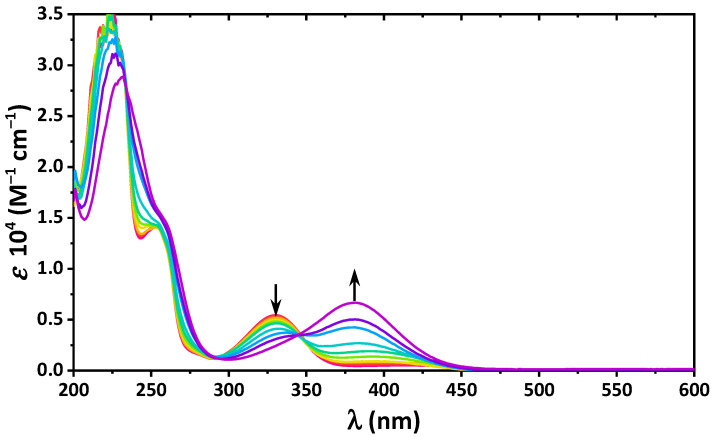
UV-vis spectra of **3** in EtOH upon gradual addition of NaOH up to 5 eqv., with a step of 0.5 eqv.

**Figure 7:**
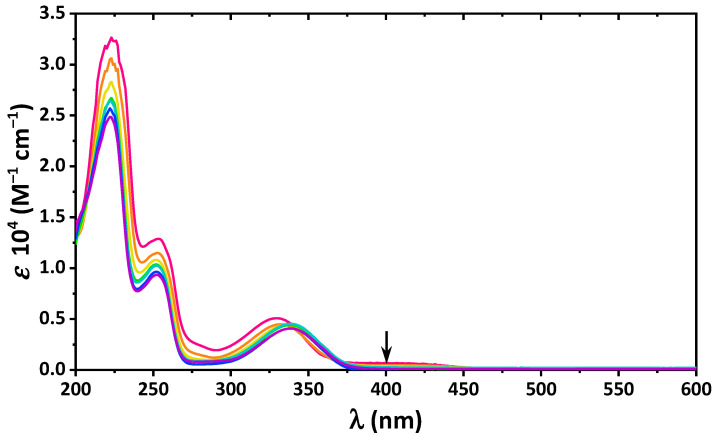
UV-vis spectra of **3** in EtOH upon gradual addition of CH_3_SO_3_H up to 3.5 eqv., with a step of 0.5 eqv.

After the addition of NaOH, the absorption band at 330 nm disappeared with the simultaneous increase of a low-intense band in the visible region (Figure 6). However, the addition of CH_3_SO_3_H to a solution of **3** in EtOH vanished the same band, arising from the *cis*-keto-enamine tautomer (Figure 7).

**Figure 8:**
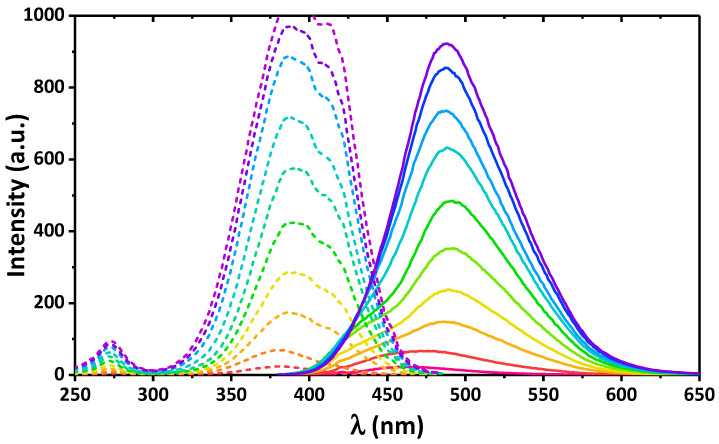
Emission (solid line) and excitation (dashed line) spectra of **3** in EtOH gradual addition of NaOH up to 5 eqv., with a step of 0.5 eqv. (λ_exc_ = 375 nm, λ_em_ = 480 nm).

Upon the addition of NaOH to a solution of **3** in EtOH, an emission band appeared (Figure 8), which deconvolution revealed two bands at 480 and 505 nm and with an area ratio of 30.1 and 69.9%, respectively (Figure S10). Based on the comparison of the absorption (Figure 6) and excitation (Figure 8) spectra, the emission band was assigned to two conformers of the deprotonated form of **3**. Solutions of **3** in EtOH were found to be non-emissive regardless of the added amount of CH_3_SO_3_H.

We have further applied the density functional theory (DFT) calculations to examine the fine features of **1**–**4**. It was established that the calculated values of bond lengths, bond angles, and dihedral angles (Table 2) were in good agreement with the values recently obtained from single-crystal X-ray diffraction [17]. The observed differences between the calculated and experimental geometrical parameters are obviously explained by the fact that the DFT computations were performed in the gas phase.

**Figure 9:**
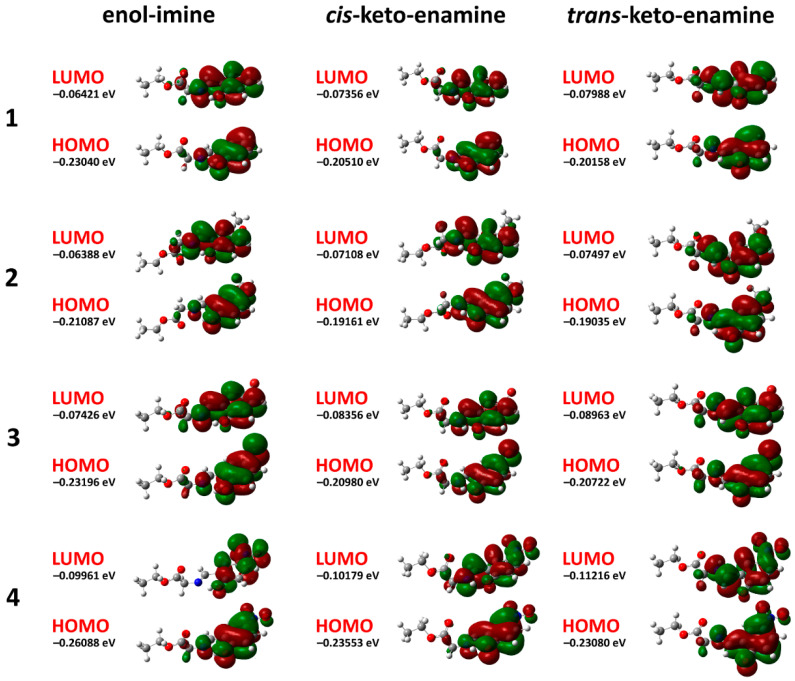
Energy levels and front views on the electronic isosurfaces of the high occupied and low unoccupied molecular orbitals of the ground state of **1–4**, obtained by using the B3LYP/6-311++G(d,p) method.

According to the DFT calculations, the dipole moments of the fully optimized ground state geometry of the enol-imine forms were 3.279813 Debye for **1**, 4.290475 Debye for **2**, 4.070018 Debye for **3**, and 6.97922 Debye for **4**. Notably, the transformation from the enol-imine to the *trans*-keto-enamine through the *cis*-keto-enamine tautomers was followed by a remarkable increase in the dipole moments for all the structures (Table 3). Significantly higher dipole moments of different tautomers of **4** in comparison to the corresponding values of tautomers of **1**–**3** were obviously explained by the presence of the highly polar NO_2_ substituent. The energies of the frontier molecular orbitals for the highest occupied molecular orbital (HOMO) and lowest-lying unoccupied molecular orbital (LUMO) are shown in Table 3. Both orbitals were mainly delocalized over the 2-OH(5-X)C_6_H_3_CH=N–CH_2_C(=O) fragment for all the structures except for the LUMO of **4**, where the orbital was mainly spread over the 2-OH(5-X)C_6_H_3_ fragment (Figure 9).

The so-called ionization potential (*I*) and the electron affinity (*A*) value of the molecules were established as follows: *I* = −*E*_HOMO_ and *A* = −*E*_LUMO_ (Table 3) [46], which determined the electron-donating ability and the ability to accept an electron, respectively. As such, as lower values of *I* as better donation of an electron, while as higher values of *A* as better ability to accept electrons. Both the *I* and *A* values for all the tautomers of **1**–**4** are remarkably lower than unity (Table 3), indicating that the reported dyes each exhibit high electron-donating and low electron-accepting properties. Notably, the corresponding *cis*-keto-enamine and *trans*-keto-enamine tautomers of **1**–**4** are slightly better electron donors and electron acceptors in comparison to their enol-imine derivatives (Table 3).

To estimate the relative reactivity of molecules of **1**–**4**, we have further established values of the so-called global chemical reactivity descriptors derived from the HOMO–LUMO energy gap (Table 3). The values of chemical potential (*μ*) for **1**–**4** were in the range from −0.13135 eV to −0.18025 eV for all tautomers, indicating the poor electron-accepting ability and the strong donating ability, which was further supported with low values of electronegativity, *χ* (Table 3). Notably, the values of electronegativity for all the *cis*-keto-enamine tautomers were lower than those for the enol-imine and *trans*-keto-enamine forms of **1**–**4** (Table 3). Chemical hardness (*η*) describes the resistance towards deformation/polarization of the electron cloud of the molecule upon a chemical reaction, while softness (*S*) is a reverse of chemical hardness [46]. Compounds **1**–**4** are characterized by low values of *η* and high values of *S*, respectively, indicating a remarkable tendency to exchange their electron clouds with the surrounding environment for all the structures (Table 3). It should be noted that the *trans*-keto-enamine tautomers are more pronounced to exchange their electron clouds with the surrounding environment in comparison to the corresponding *cis*-keto-enamine tautomers, and even much more pronounced in comparison to the enol-imine tautomers (Table 3). The electrophilicity index (*ω*) describes the energy of stabilization to accept electrons [46]. The *ω* values for all forms of **1**–**4** were found in the range from about 0.12 eV to 0.25 eV (Table 3). These values are low, indicating the strong nucleophilic nature of **1–4**. Finally, compounds **1**–**4** can accept about 1.77–2.24 electrons for the enol-imine forms, 2.12–2.52 electrons for the *cis*-keto-enamine forms, and 2.30–2.89 electrons for the *trans*-keto-enamine forms, respectively, as evidenced from the ΔN_max_ values, of which the highest values correspond to **4** (Table 3).

**Figure 10:**
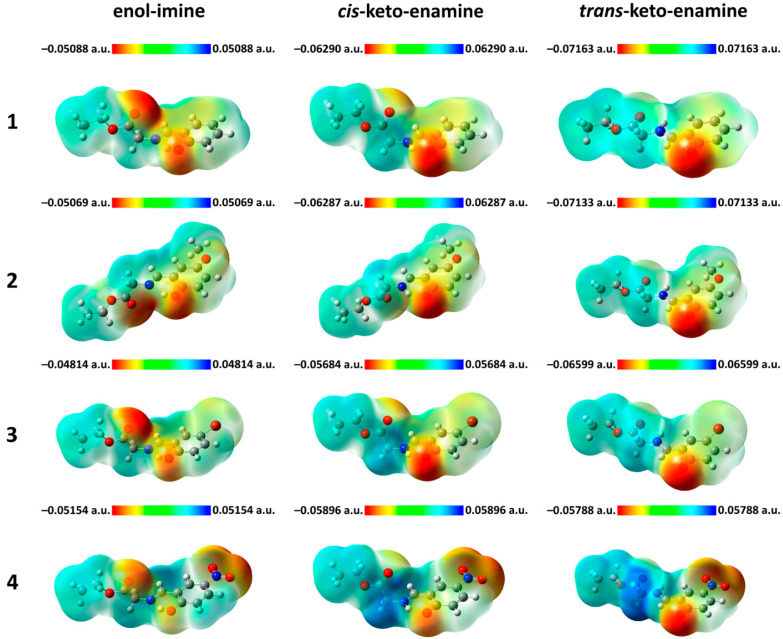
Views of the molecular electrostatic potential surface of **1**–**4**, obtained by using the B3LYP/6-311++G(d,p) method.

The electrophilic and nucleophilic sites in **1**–**4** were examined using the molecular electrostatic potential (MEP) analysis. The red and blue colors of the MEP surface correspond to electron-rich (nucleophilic) and electron-deficient (electrophilic) regions, respectively. On the MEP surfaces of the enol-imine tautomers of **1**–**4** the most pronounced nucleophilic centers are located on the carbonyl and hydroxyl oxygen atoms, while the other negative electrostatic potential sites in the enol-imine form of **4** are located on the oxygen atoms of the NO_2_ group (Figure 10). In the *cis*-keto-enamine and *trans*-keto-enamine tautomers of **1**–**4** the carbonyl oxygen atom attached to the aromatic ring is the most remarkable nucleophilic center, alongside with both oxygen atoms of the NO_2_ group in **4**, while the carbonyl oxygen atom of the carboxyl group becomes a less pronounced nucleophilic center (Figure 10). As the most electrophilic region the CH=N–CH_2_ fragment for the enol-imine tautomers and the CH–NH–CH_2_ fragment for the *cis*-keto-enamine and *trans*-keto-enamine tautomers can be highlighted for the structures of **1**–**4** (Figure 10).

The calculated absorption spectra of the fully optimized ground state geometry of all the three tautomers of **1**–**4** (Figures S11–S13) are in agreement with experimental spectra. In particular, the experimental UV-vis spectra for the enol-imine tautomers exhibited bands at 215–260 and 331–353 nm, and the calculated spectra for the same tautomers contained bands at 225–250 and 302–351 nm (Table S1). The *cis*-keto-enamine tautomers are shown as a band centered at 382–418 nm in both the experimental and calculated absorption spectra (Table S1). The calculated UV-vis spectra for the *trans*-keto-enamine tautomers of **1**–**4** each exhibited a low-energy band at 407–428 nm, while no similar bands were observed in the corresponding experimental spectra (Table S1), thus, testifying to the absence of the *trans*-keto-enamine tautomers of **1**–**4** in the applied solvents.

The calculated UV-vis spectra of the enol-imine forms of **1**–**4** exhibit absorption bands with three (for **1**) or two (for **2**–**4**) maxima exclusively in the UV region centered at about 220–255 nm and 300–350 nm (Figure S11). These bands mostly corresponded to the HOMO–1 → LUMO, HOMO → LUMO, and HOMO → LUMO+3 transitions (Table 4, Figure S11). The absorption bands in the spectra of **3** and **4** were additionally supported by the HOMO → LUMO+1 transition and further described by the HOMO–4 → LUMO transition in the spectrum of **3**, and by the HOMO–3 → LUMO+1 and HOMO–1 → LUMO+1 transitions in the spectrum of **4**, respectively (Table 4, Figure S11). As for the calculated UV-vis spectra of the *cis*-keto-enamine and *trans*-keto-enamine tautomers of **1–4**, absorption bands are observed in both the UV and visible regions up to about 500–550 nm (Figure S12) and 600 nm (Figure S13), respectively. These bands are characterized by two maxima centered at about 250–290 nm and 380–430 nm (Figures S12 and S13). Notably, the calculated UV-vis spectrum of the *cis*-keto-enamine form of **4** contained an additional maxima at 320 nm (Figure S12). The corresponding transitions, responsible for the observed bands in the calculated UV-vis spectra of the *cis*-keto-enamine and *trans*-keto-enamine tautomers of **1**–**4**, are shown in Figures S14–S17 and collected in Tables S2 and S3.

## 3. Materials and Methods

### 3.1. Materials

All reagents and solvents were commercially available and used without further purification. Dyes **1**–**4** were obtained according to the described synthetic procedure [18].

### 3.2. Physical Measurements

Absorption and fluorescent spectra from the freshly prepared solutions (10^−4^ M) in freshly distilled solvents were recorded on an Agilent 8453 instrument.

### 3.3. Computational Details

The ground state geometry of **1**–**4** was fully optimized without symmetry restrictions. The calculations were performed by means of the GaussView 6.0 molecular visualization program [47] and Gaussian 09, Revision D.01 program package [48] using the density functional theory (DFT) method with Becke three-parameter Lee-Yang-Parr (B3LYP) hybrid functional [49,50] and 6-311++G(d,p) [49,51] basis set. The crystal structure geometry was used as a starting model for structural optimization. The vibration frequencies were calculated for the optimized structures in the gas phase and no imaginary frequencies were obtained. The Cartesian atomic coordinates for the optimized structures are gathered in Tables S4–S7. The electronic isosurfaces of the HOMO and LUMO orbitals and MEP surfaces of all tautomers of **1**–**4** were generated from the fully optimized ground state geometry obtained by using the B3LYP/6-311++G(d,p) method. The absorption spectra of the fully optimized ground state geometry of all the three tautomers of **1–4** were simulated at the TD-DFT/B3LYP/6-311++G(d,p) level.

## 4. Conclusions

In summary, herein we discuss structural studies using the Hirshfeld molecular surface approach, as well as photophysical properties of four dyes: ethyl *N*-salicylideneglycinate (**1**), ethyl *N*-(5-methoxysalicylidene)glycinate (**2**), ethyl *N*-(5-bromosalicylidene)glycinate (**3**), and ethyl *N*-(5-nitrosalicylidene)glycinate (**4**).

The intermolecular H···H, H···C, and H···O contacts are the dominant contributors to the molecular surface of all the reported compounds. **3** is further described by a remarkable proportion of the intermolecular H···Br contacts. Furthermore, a distinct proportion of the C∙∙∙C contacts was found in the molecular surface of **3** and **4**, which is explained by intermolecular π⋯π stacking interactions between the phenylene rings.

The absorption spectra of **4** in THF and CH_3_CN exhibit a band for the *cis*-keto-enamine form, while only the enol-imine tautomer was found in the absorption spectrum of **4** in cyclohexane and in the absorption spectra of **1**–**3** in cyclohexane, THF, and CH_3_CN. Furthermore, in the absorption spectra of **4** in MeOH, EtOH, *i*PrOH, and *n*BuOH the *cis*-keto-enamine form is clearly observed, with the most remarkable one in MeOH, while the same band is absent in the spectrum of **4** in *n*PrOH, that might be explained by possible specific solute-solvent interactions. Dye **4** is emissive in *i*PrOH and *n*PrOH, arising from two conformers of the *cis*-keto-enamine* form and from the *trans*-keto-enamine* form, while the origin of emission in *n*PrOH is exclusively from different *cis*-keto-enamine* conformers. Titration of the solutions of **3** and **4** in EtOH by NaOH leads to the gradual increasing of the band in the visible region of the corresponding UV-vis spectra. The same band gradually decreases and finally vanishes upon titration of the solutions of **3** and **4** in EtOH by CH_3_SO_3_H due to the protonation of the imine N-atom. Notably, upon addition of NaOH to the solution of **3** in EtOH, an emission band appeared and increased with increasing NaOH concentration.

We have also demonstrated that photophysical properties of a reported series of closely related compounds **1**–**4** can efficiently be tuned not only by changing the corresponding substituent in the phenolic ring, thus changing electronic properties, but also by the nature of a solvent (non-polar vs. polar aprotic vs. polar protic) and pH (NEt_3_ or NaOH, and CH_3_SO_3_H). All these factors allow to fine influence to the keto-enamine–enol-imine tautomerization both in the ground and excited states, yielding two or even three emission bands with a certain ratio and, as a result, different resulting emission color. No doubt, all these findings are of potential interest for molecular optics. Furthermore, the presence of the ethyl glycinate fragment in **1–4** might play a pivotal role for the application of these compounds, e.g., as luminescent sensors, in biological systems.

According to the DFT calculation results, it was established that all the tautomers of **1**–**4** each exhibit high electron-donating and low electron-accepting properties. The most pronounced nucleophilic centers of the enol-imine tautomers are located on the carbonyl and hydroxyl oxygen atoms, while the other negative electrostatic potential sites in the enol-imine form of **4** are located on the oxygen atoms of the NO_2_ group. In the *cis*-keto-enamine and *trans*-keto-enamine tautomers of **1**–**4**, the carbonyl oxygen atom attached to the aromatic ring is the most remarkable nucleophilic center, alongside both oxygen atoms of the NO_2_ group in **4**, while the carbonyl oxygen atom of the carboxyl group becomes a less pronounced nucleophilic center. As the most electrophilic region, the CH=N–CH_2_ fragment for the enol-imine tautomers and the CH–NH–CH_2_ fragment for the *cis*-keto-enamine and *trans*-keto-enamine tautomers are highlighted for the structures of **1**–**4**. The calculated absorption spectra of the fully optimized ground state geometry of all the three tautomers of **1**–**4** are in good agreement with experimental spectra.

## Figures and Tables

**Table 1:** Hirshfeld contact surfaces and derived “random contacts” and “enrichment ratios” for the crystal structures of **1–4**.

	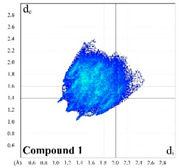				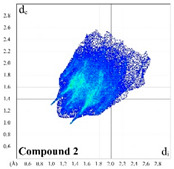				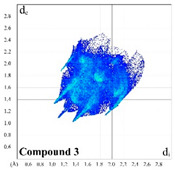					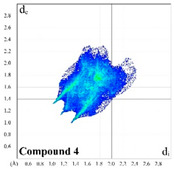			
	H	C	N	O	H	C	N	O	H	C	N	O	Br	H	C	N	O
Contacts (*C*, %) ^1^																	
H	48.3	–	–	–	45.5	–	–	–	35.4	–	–	–	–	30.0	–	–	–
C	22.6	0.0	–	–	20.9	0.6	–	–	13.7	4.1	–	–	–	11.5	4.9	–	–
N	2.4	0.0	0.0	–	2.4	0.0	0.0	–	1.9	0.4	0.0	–	–	3.3	0.6	0.0	–
O	24.9	1.2	0.0	0.6	30.1	0.3	0.0	0.3	23.4	1.6	0.5	0.2	–	44.1	1.8	1.7	2.1
Br	–	–	–	–	–	–	–	–	17.4	0.5	0.0	0.0	0.9	–	–	–	–
Surface (*S*, %)																	
	73.3	11.9	1.2	13.7	72.2	11.2	1.2	15.5	63.6	12.2	1.4	13.0	9.9	59.5	11.9	2.8	25.9
Random contacts (*R*, %)																	
H	53.7	–	–	–	52.1	–	–	–	40.4	–	–	–	–	35.4	–	–	–
C	17.4	1.4	–	–	16.2	1.3	–	–	15.5	1.5	–	–	–	14.2	1.4	–	–
N	1.8	0.0	0.0	–	1.7	0.0	0.0	–	1.8	0.3	0.0	–	–	3.3	0.0	0.1	–
O	20.1	3.3	0.3	1.9	22.4	3.5	0.4	2.4	16.5	3.2	0.4	1.7	–	30.8	6.2	1.5	6.7
Br	–	–	–	–	–	–	–	–	12.6	2.4	0.3	2.6	1.0	–	–	–	–
Enrichment (*E*) ^2^																	
H	0.90	–	–	–	0.87	–	–	–	0.88	–	–	–	–	0.85	–	–	–
C	1.30	0.0	–	–	1.29	0.46	–	–	0.88	2.73	–	–	–	0.81	3.50	–	–
N	1.33	–	–	–	1.41	–	–	–	1.06	–	–	–	–	1.00	–	–	–
O	1.24	0.36	–	0.32	1.34	0.09	–	0.13	1.42	0.50	–	0.12	–	1.43	0.29	1.13	0.31
Br	–	–	–	–	–	–	–	–	1.38	0.21	–	0.0	0.90	–	–	–	–

^1^ Values are obtained from CrystalExplorer 3.1 [41]. ^2^ The “enrichment ratios” were not computed when the “random contacts” were lower than 0.9%, as they are not meaningful [42].

**Table 2:** Selected bond lengths (Å) and angles (°) in the structures of **1–4**, obtained by using the B3LYP/6-311++G(d,p) method ^1^.

	1	2	3	4
Bond lengths				
C=N	1.2816	1.2821	1.2800	1.2787
C–O(H)	1.3410	1.3448	1.3390	1.3298
C=O	1.2034	1.2036	1.2030	1.2031
C–O(Et)	1.3462	1.3461	1.8529	1.3440
Bond angles				
C=N–C	119.04	118.98	119.17	119.43
Dihedral angles ^2^				
N–C–C=O	29.12	−31.15	24.53	21.83
O=C–O–C	1.54	−1.44	1.53	1.46
C–O–C–C	−179.86	179.95	−0.21	179.87
Hydrogen bonds				
O–H	0.9927	0.9913	0.9933	0.9985
H∙∙∙N	1.7472	1.7546	1.7464	1.7200
O∙∙∙N	5.6362	2.6395	2.6342	2.6160
∠(O–H∙∙∙N)	147.05	146.66	146.79	147.22

^1^ The computational results have been compared according to the crystallographic data [17]. ^2^ The corresponding dihedral angles must be compared by their magnitudes.

**Table 3:** Total energy, dipole moment, frontier molecular HOMO and LUMO orbitals, gap value, and descriptors for **1–4** in gas phase, obtained by using the B3LYP/6-311++G(d,p) method.

	1	2	3	4
enol-imine tautomer				
Total energy (eV)	−19,255.75967	−22,372.91079	−89,285.41965	−24,822.27922
Dipole moment (Debye)	3.279813	4.290475	4.070018	6.97922
*E*_HOMO_ (eV)	−0.23040	−0.21087	−0.23196	−0.26088
*E*_LUMO_ (eV)	−0.06421	−0.06388	−0.07426	−0.09961
Δ*E*_LUOMO–HOMO_ = *E*_LUMO_−*E*_HOMO_ (eV)	0.16619	0.14699	0.15770	0.16127
Ionization energy, *I* = −*E*_HOMO_ (eV)	0.23040	0.21087	0.23196	0.26088
Electron affinity, *A* = −*E*_LUMO_ (eV)	0.06421	0.06388	0.07426	0.09961
Electronegativity, *χ* = (*I* + *A*)/2 (eV)	0.14731	0.13738	0.15311	0.18025
Chemical potential, *μ* = −*χ* (eV)	−0.14731	−0.13738	−0.15311	−0.18025
Global chemical hardness, *η* = (*I−A*)/2 (eV)	0.08310	0.07350	0.07885	0.08064
Global chemical softness, *S* = 1/(2*η*) (eV^−1^)	6.01721	6.80318	6.34115	6.20078
Global electrophilicity index, *ω* = *μ*^2^/(2*η*) (eV)	0.13057	0.12839	0.14865	0.20145
Global nucleophilicity index, *E* = *μ* × *η* (eV^2^)	−0.01224	−0.01010	−0.01207	−0.01453
Maximum additional electric charge, ΔN_max_ = −*μ*/*ƞ*	1.77273	1.86917	1.94179	2.23532
*cis*-keto-enamine tautomer				
Total energy (eV)	−19,255.55920	−22,372.72632	−89,285.24115	−24,822.17304
Dipole moment (Debye)	5.623354	6.171161	6.678792	10.089039
*E*_HOMO_ (eV)	−0.20510	−0.19161	−0.20980	−0.23553
*E*_LUMO_ (eV)	−0.07356	−0.07108	−0.08356	−0.10179
Δ*E*_LUOMO–HOMO_ = *E*_LUMO_−*E*_HOMO_ (eV)	0.13154	0.12053	0.12624	0.13374
Ionization energy, *I* = −*E*_HOMO_ (eV)	0.20510	0.19161	0.20980	0.23553
Electron affinity, *A* = −*E*_LUMO_ (eV)	0.07356	0.07108	0.08356	0.10179
Electronegativity, *χ* = (*I* + *A*)/2 (eV)	0.13933	0.13135	0.14668	0.16866
Chemical potential, *μ* = −*χ* (eV)	−0.13933	−0.13135	−0.14668	−0.16866
Global chemical hardness, *η* = (*I*−*A*)/2 (eV)	0.06577	0.06027	0.06312	0.06687
Global chemical softness, *S* = 1/(2*η*) (eV^−1^)	7.60225	8.29669	7.92142	7.47719
Global electrophilicity index, *ω* = *μ*^2^/(2*η*) (eV)	0.14758	0.14313	0.17043	0.21270
Global nucleophilicity index, *E* = *μ* × *η* (eV^2^)	−0.00916	−0.00792	−0.00926	−0.01128
Maximum additional electric charge, ΔN_max_ = −*μ*/*ƞ*	2.11844	2.17946	2.32383	2.52221
*trans*-keto-enamine tautomer				
Total energy (eV)	−19,255.22025	−22,372.44811	−89,284.91121	−24,821.82359
Dipole moment (Debye)	7.396322	7.781535	7.644000	10.221661
*E*_HOMO_ (eV)	−0.20158	−0.19035	−0.20722	−0.23080
*E*_LUMO_ (eV)	−0.07988	−0.07497	−0.08963	−0.11216
Δ*E*_LUOMO–HOMO_ = *E*_LUMO_−*E*_HOMO_ (eV)	0.12170	0.11538	0.11759	0.11864
Ionization energy, *I* = −*E*_HOMO_ (eV)	0.20158	0.19035	0.20722	0.23080
Electron affinity, *A* = −*E*_LUMO_ (eV)	0.07988	0.07497	0.08963	0.11216
Electronegativity, *χ* = (*I* + *A*)/2 (eV)	0.14073	0.13266	0.14843	0.17148
Chemical potential, *μ* = −*χ* (eV)	−0.14073	−0.13266	−0.14843	−0.17148
Global chemical hardness, *η* = (*I*−*A*)/2 (eV)	0.06085	0.05769	0.05880	0.05932
Global chemical softness, *S* = 1/(2*η*) (eV^−1^)	8.21693	8.66701	8.50412	8.42886
Global electrophilicity index, *ω* = *μ*^2^/(2*η*) (eV)	0.16274	0.15253	0.18735	0.24785
Global nucleophilicity index, *E* = *μ* × *η* (eV^2^)	−0.00856	−0.00765	−0.00873	−0.01017
Maximum additional electric charge, ΔN_max_ = –*μ*/*ƞ*	2.31274	2.29953	2.52445	2.89076

**Table 4:** Values for the calculated UV-vis spectra of the ground state of the enol-imine tautomers of **1**–**4**, obtained by using the TD-DFT/B3LYP/6-311++G(d,p) method.

**1**			**2**		
λ_max_ (nm)	Oscillator strength	Transitions	λ_max_ (nm)	Oscillator strength	Transitions
220.6	0.1419	HOMO–3 → LUMO (14.0%)	239.1	0.2994	HOMO–3 → LUMO (9.0%)
		HOMO–1 → LUMO (8.1%)			HOMO–1 → LUMO (26.8%)
		HOMO–1 → LUMO+3 (2.5%)			HOMO → LUMO+3 (50.2%)
		HOMO–1 → LUMO+9 (2.0%)			HOMO → LUMO+10 (3.4%)
		HOMO → LUMO+1 (2.7%)			HOMO → LUMO+13 (2.3%)
		HOMO → LUMO+2 (10.7%)			
		HOMO → LUMO+3 (44.7%)			
		HOMO → LUMO+9 (7.2%)			
222.6	0.0311	HOMO → LUMO+1 (5.6%)	244.3	0.0188	HOMO–1 → LUMO (2.8%)
		HOMO → LUMO+2 (45.0%)			HOMO → LUMO+1 (3.7%)
		HOMO → LUMO+3 (24.2%)			HOMO → LUMO+2 (85.9%)
		HOMO → LUMO+4 (8.6%)			HOMO → LUMO+5 (3.9%)
		HOMO → LUMO+5 (7.5%)			
228.5	0.0315	HOMO–3 → LUMO (70.8%)	251.2	0.0720	HOMO–1 → LUMO (21.3%)
		HOMO–3 → LUMO+1 (4.0%)			HOMO → LUMO+1 (7.1%)
		HOMO–3 → LUMO+3 (2.4%)			HOMO → LUMO+3 (45.5%)
		HOMO → LUMO+2 (12.3%)			HOMO → LUMO+4 (23.2%)
		HOMO → LUMO+3 (3.5%)			
252.8	0.2896	HOMO–1 → LUMO (76.5%)	256.6	0.0742	HOMO–1 → LUMO (33.4%)
		HOMO → LUMO (3.2%)			HOMO → LUMO+3 (49.0%)
		HOMO → LUMO+1 (4.7%)			HOMO → LUMO+4 (10.9%)
		HOMO → LUMO+2 (9.8%)			
312.1	0.1055	HOMO–1 → LUMO (2.3%)	350.5	0.1086	HOMO → LUMO (96.9%)
		HOMO → LUMO (93.9%)			
**3**			**4**		
λ_max_ (nm)	Oscillator strength	Transitions	λ_max_ (nm)	Oscillator strength	Transitions
224.7	0.0487	HOMO–5 → LUMO (10.6%)	240.4	0.0556	HOMO–3 → LUMO (85.4%)
		HOMO → LUMO+4 (57.1%)			HOMO–1 → LUMO+1 (9.6%)
		HOMO → LUMO+5 (23.2%)			
228.4	0.2498	HOMO–5 → LUMO (3.1%)	244.8	0.2633	HOMO–3 → LUMO (8.1%)
		HOMO–4 → LUMO (32.2%)			HOMO–3 → LUMO+1 (29.7%)
		HOMO–1 → LUMO (16.9%)			HOMO–1 → LUMO+1 (55.7%)
		HOMO → LUMO+1 (17.2%)			
		HOMO → LUMO+2 (6.0%)			
		HOMO → LUMO+3 (7.5%)			
		HOMO → LUMO+5 (2.3%)			
		HOMO → LUMO+8 (3.9%)			
237.8	0.2157	HOMO–4 → LUMO (28.8%)	273.4	0.1054	HOMO–1 → LUMO (77.0%)
		HOMO–3 → LUMO (2.9%)			HOMO → LUMO+1 (18.3%)
		HOMO–1 → LUMO (16.3%)			
		HOMO → LUMO+1 (32.7%)			
		HOMO → LUMO+2 (2.6%)			
		HOMO → LUMO+3 (9.3%)			
		HOMO → LUMO+4 (2.1%)			
252.7	0.1308	HOMO–1 → LUMO (57.9%)	299.4	0.0744	HOMO–1 → LUMO (13.6%)
		HOMO → LUMO+1 (35.1%)			HOMO → LUMO (34.4%)
					HOMO → LUMO+1 (48.9%)
329.3	0.0858	HOMO → LUMO (95.7%)	308.7	0.2162	HOMO–1 → LUMO (5.1%)
					HOMO–1 → LUMO+1 (3.1%)
					HOMO → LUMO (61.8%)
					HOMO → LUMO+1 (28.9%)

## Data Availability

Not available.

## Supplementary Materials

The following are available online, Figures S1–S4: 2D and decomposed 2D fingerprint plots of observed contacts for the crystal structure of **1–4**, Figures S5–S7: UV-vis spectra of **1–3** in the applied solvents, Figure S8: Emission and excitation spectra of **4** in *n*PrOH and *i*PrOH, Figure S9: UV-vis spectra of **4** in EtOH upon gradual addition of NEt_3_, Figure S10: Emission and excitation spectra of **3** in EtOH after addition of 5 eqv. of NaOH, Figure S11: The calculated UV-vis spectra of the ground states of the enol-imine tautomers of **1**–**4**, Figure S12: The calculated UV-vis spectra of the ground states of the *cis*-keto-enamine tautomers of **1**–**4**, Figure S13: The calculated UV-vis spectra of the ground states of the *trans*-keto-enamine tautomers of **1**–**3**, Figures S14–S17: Energy levels and views on the electronic isosurfaces of the selected molecular orbitals of the ground state of **1**–**4**, Table S1: Values for the main maxima in the experimental UV-vis spectra of **1–4** in different solvents, and in the calculated UV-vis spectra for different tautomers of **1**–**4**, Table S2: Values for the calculated UV-vis spectra of the ground state for the *cis*-keto-enamine tautomers of **1**–**4**, Table S3: Values for the calculated UV-vis spectra of the ground state for the *trans*-keto-enamine tautomers of **1**–**4**, Tables S4–S7: Cartesian atomic coordinates for optimized structures of **1**–**4**, obtained by using the DFT/B3LYP/6–311++G(d,p) method.

## Sample Availability

Samples of the compounds ethyl *N*-salicylideneglycinate (**1**), ethyl *N*-(5-methoxysalicylidene)glycinate (**2**), ethyl *N*-(5-bromosalicylidene)glycinate (**3**), and ethyl *N*-(5-nitrosalicylidene)glycinate (**4**) are available from the authors.

## References

[1] Sacconi L. (1996). Tetrahedral complexes of nickel (II) and copper (II) with schiff bases. Coord. Chem. Rev..

[2] Yamada S. (1966). Recent aspects of the stereochemistry of schiff-base-metal complexes. Coord. Chem. Rev..

[3] Nath M., Goyal S. (1996). Organotin (Iv) complexes of schiff bases: A review. Main Group Met. Chem..

[4] Aggarwal N., Kumar R., Dureja P., Diwan S., Rawat D.S. (2009). Schiff bases as potential fungicides and nitrification inhibitors. J. Agric. Food Chem..

[5] Weng Q., Yi J., Chen X., Luo D., Wang Y., Sun W., Kang J., Han Z. (2020). Controllable Synthesis and Biological Application of Schiff Bases from d-Glucosamine and Terephthalaldehyde. ACS Omega.

[6] Ferraz de Paiva R.E., Marçal Neto A., Santos I.A., Jardim A.C.G., Corbi P.P., Bergamini F.R.G. (2020). What is holding back the development of antiviral metallodrugs? A literature overview and implications for SARS-CoV-2 therapeutics and future viral outbreaks. Dalton Trans..

[7] Wang P.H., Keck G.J., Lien E.J., Lai M.M.C. (1990). Design, Synthesis, Testing, and Quantitative Structure-Activity Relationship Analysis of Substituted Salicylaldehyde Schiff Bases of 1-Amino-3-Hydroxyguani-dine Tosylate as New Antiviral Agents against Coronavirus. J. Med. Chem..

[8] Safin D.A., Robeyns K., Garcia Y. (2012). Crown ether-containing *N*-salicylidene aniline derivatives: Synthesis, characterization and optical properties. CrystEngComm.

[9] Safin D.A., Robeyns K., Garcia Y. (2012). Solid-state thermo- and photochromism in *N*,*N′*-bis(5-X-salicylidene)diamines (X = H, Br). RSC Adv..

[10] Safin D.A., Garcia Y. (2013). First evidence of thermo- and two-step photochromism of tris-anils. RSC Adv..

[11] Safin D.A., Bolte M., Garcia Y. (2014). Photoreversible solid state negative photochromism of *N*-(3,5-dichlorosalicylidene)-1-aminopyrene. CrystEngComm.

[12] Safin D.A., Babashkina M.G., Robeyns K., Bolte M., Garcia Y. (2014). *N*-Salicylidene aniline derivatives based on the *N′*-thiophosphorylated thiourea scaffold. CrystEngComm.

[13] Safin D.A., Bolte M., Garcia Y. (2014). Solid-state photochromism and thermochromism of *N*-salicylidene pyrene derivatives. CrystEngComm.

[14] Safin D.A., Babashkina M.G., Robeyns K., Garcia Y. (2016). C–H⋯Br–C vs. C–Br⋯Br–C vs. C–Br⋯N bonding in molecular self-assembly of pyridine-containing dyes. RSC Adv..

[15] Safin D.A., Robeyns K., Babashkina M.G., Filinchuk Y., Rotaru A., Jureschi C., Mitoraj M.P., Hooper J., Brela M., Garcia Y. (2016). Polymorphism driven optical properties of an anil dye. CrystEngComm.

[16] Safin D.A., Robeyns K., Garcia Y. (2016). 1,2,4-Triazole-based molecular switches: Crystal structures, Hirshfeld surface analysis and optical properties. CrystEngComm.

[17] Shapenova D.S., Shiryaev A.A., Bolte M., Kukułka M., Szczepanik D.W., Hooper J., Babashkina M.G., Mahmoudi G., Mitoraj M.P., Safin D.A. (2020). Resonance Assisted Hydrogen Bonding Phenomenon Unveiled from Both Experiment and Theory–an Example of New Family of Ethyl *N*-salicylideneglycinate Dyes. Chem. Eur. J..

[18] Shiryaev A.A., Burkhanova T.M., Mahmoudi G., Babashkina M.G., Safin D.A. (2020). Photophysical properties of ethyl *N*-(5-bromosalicylidene)glycinate and ethyl *N*-(5-nitrosalicylidene)glycinate in CH_2_Cl_2_. J. Lumin..

[19] Hadjoudis E., Vittorakis M., Moustakali-Mavridis I. (1987). Photochromism and thermochromism of schiff bases in the solid state and in rigid glasses. Tetrahedron.

[20] Hadjoudis E., Mavridis I.M. (2004). Photochromism and thermochromism of Schiff bases in the solid state: Structural aspects. Chem. Soc. Rev..

[21] Amimoto K., Kawato T. (2005). Photochromism of organic compounds in the crystal state. J. Photochem. Photobiol. C.

[22] Haneda T., Kawano M., Kojima T., Fujita M. (2007). Thermo-to-photo-switching of the chromic behavior of salicylideneanilines by inclusion in a porous coordination network. Angew. Chem. Int. Ed..

[23] Filarowski A., Koll A., Sobczyk L. (2009). Intramolecular Hydrogen Bonding in o-hydroxy Aryl Schiff Bases. Curr. Org. Chem..

[24] Bertolasi V., Gilli P., Gilli G. (2009). Crystal Chemistry and Prototropic Tautomerism in 2-(1-Iminoalkyl)- phenols (or naphthols) and 2-Diazenyl-phenols (or naphthols). Curr. Org. Chem..

[25] Hadjoudis E., Chatziefthimiou S.D., Mavridis I.M. (2009). Anils: Photochromism by H-transfer. Curr. Org. Chem..

[26] Minkin V.I., Tsukanov A.V., Dubonosov A.D., Bren V.A. (2011). Tautomeric Schiff bases: Iono-, solvato-, thermo-and photochromism. J. Mol. Struct..

[27] Inokuma Y., Kawano M., Fujita M. (2011). Crystalline molecular flasks. Nat. Chem..

[28] Mahmudov K.T., Pombeiro A.J.L. (2016). Resonance-Assisted Hydrogen Bonding as a Driving Force in Synthesis and a Synthon in the Design of Materials. Chem. Eur. J..

[29] Jana S., Dalapati S., Guchhait N. (2012). Proton Transfer Assisted Charge Transfer Phenomena in Photochromic Schiff Bases and Effect of–NEt2 Groups to the Anil Schiff Bases. J. Phys. Chem. A.

[30] Germino J.C., Barboza C.A., Quites F.J., Vazquez P.A.M., Atvars T.D.Z. (2015). Dual Emissions of Salicylidene-5-chloroaminepyridine Due to Excited State Intramolecular Proton Transfer: Dynamic Photophysical and Theoretical Studies. J. Phys. Chem. C.

[31] Zhao L., Sui D., Chai J., Wang Y., Jiang S. (2006). Digital Logic Circuit Based on a Single Molecular System of Salicylidene Schiff Base. J. Phys. Chem. B.

[32] Kleij A.W. (2008). New Templating Strategies with Salen Scaffolds (Salen = *N*,*N′*-Bis(salicylidene)ethylenediamine Dianion). Chem. Eur. J..

[33] Cheng J., Ma X., Zhang Y., Liu J., Zhou X., Xiang H. (2014). Optical Chemosensors Based on Transmetalation of Salen-Based Schiff Base Complexes. Inorg. Chem..

[34] Martín C., Fiorani G., Kleij A.W. (2015). Recent Advances in the Catalytic Preparation of Cyclic Organic Carbonates. ACS Catal..

[35] Fiorani G., Guo W., Kleij A.W. (2015). Sustainable conversion of carbon dioxide: The advent of organocatalysis. Green Chem..

[36] Beens H., Grellmann K.H., Gurr M., Weller A.H. (1965). Effect of solvent and temperature on proton transfer reactions of excited molecules. Discuss. Faraday Soc..

[37] Zhao J., Ji S., Chen Y., Guo H., Yang P. (2012). Excited state intramolecular proton transfer (ESIPT): From principal photophysics to the development of new chromophores and applications in fluorescent molecular probes and luminescent materials. Phys. Chem. Chem. Phys..

[38] Padalkar V.S., Seki S. (2016). Excited-state intramolecular proton-transfer (ESIPT)-inspired solid state emitters. Chem. Soc. Rev..

[39] Spackman M.A., Jayatilaka D. (2009). Hirshfeld surface analysis. CrystEngComm.

[40] Spackman M.A., McKinnon J.J. (2002). Fingerprinting intermolecular interactions in molecular crystals. CrystEngComm.

[41] Wolff S.K., Grimwood D.J., McKinnon J.J., Turner M.J., Jayatilaka D., Spackman M.A. (2012). CrystalExplorer 3.1.

[42] Jelsch C., Ejsmont K., Huder L. (2014). The enrichment ratio of atomic contacts in crystals, an indicator derived from the Hirshfeld surface analysis. IUCrJ.

[43] Alarcón S.H., Olivieri A.C., Sanz D., Claramunt R.M., Elguero J. (2004). Substituent and solvent effects on the proton transfer equilibrium in anils and azo derivatives of naphthol. Multinuclear NMR study and theoretical calculations. J. Mol. Struct..

[44] Nagy P.I., Fabian W.M.F. (2006). Theoretical Study of the Enol Imine ↔ Enaminone Tautomeric Equilibrium in Organic Solvents. J. Phys. Chem. B.

[45] Krygowski T.M., Woźniak K., Anulewicz R., Pawlak D., Kolodziejski W., Grech E., Szady A. (1997). Through-Resonance Assisted Ionic Hydrogen Bonding in 5-Nitro-N-salicylideneethylamine. J. Phys. Chem. A.

[46] Geerlings P., De Proft F., Langenaeker W. (2003). Conceptual Density Functional Theory. Chem. Rev..

[47] Dennington R., Keith T.A., Millam J.M. (2016). GaussView, Version 6.0.

[48] Frisch M.J., Trucks G.W., Schlegel H.B., Scuseria G.E., Robb M.A., Cheeseman J.R., Scalmani G., Barone V., Mennucci B., Petersson G.A. (2016). Gaussian 09, Revision D.01.

[49] Krishnan R., Binkley J.S., Seeger R., Pople J.A. (1980). Self-consistent molecular orbital methods. XX. A basis set for correlated wave functions. J. Chem. Phys..

[50] Becke A.D. (1993). Density-functional thermochemistry. III. The role of exact exchange. J. Chem. Phys..

[51] Frisch M.J., Pople J.A., Binkley J.S. (1984). Self-consistent molecular orbital methods 25. Supplementary functions for Gaussian basis sets. J. Chem. Phys..

