# Supplementary material for: A Family of Ethyl N-Salicylideneglycinate Dyes Stabilized by Intramolecular Hydrogen Bonding: Photophysical Properties and Computational Study
Source: Molecules. 2021 May 23;26(11):3112. doi: 10.3390/molecules26113112 (PMC8197002; doi:10.3390/molecules26113112)
Supplement: Supplementary file 1 [file molecules-26-03112-s001.zip › molecules-1214822-supplementary.pdf]

# Supplementary Information

# A Family of Ethyl *N*-salicylideneglycinate Dyes Stabilized by Intramolecular Hydrogen Bonding: Photophysical Properties and Computational Study

**Larisa E. Alkhimova [1], Maria G. Babashkina [2] and Damir A. Safin [1,3,4,*]**

[1] University of Tyumen, Volodarskogo Str. 6, 625003 Tyumen, Russia
[2] Institute of Condensed Matter and Nanosciences, Université Catholique de Louvain, Place L. Pasteur 1, 1348 Louvain-la-Neuve, Belgium; maria.babashkina@gmail.com
[3] Kurgan State University, Sovetskaya Str. 63/4, Kurgan 640020, Russia
[4] Innovation Center for Chemical and Pharmaceutical Technologies, Ural Federal University named after the First President of Russia B.N. Eltsin, Mira Str. 19, Ekaterinburg 620002, Russian Federation
* Correspondence: l.e.alkhim@gmail.com (L.E.A.); damir.a.safin@gmail.com (D.A.S.)

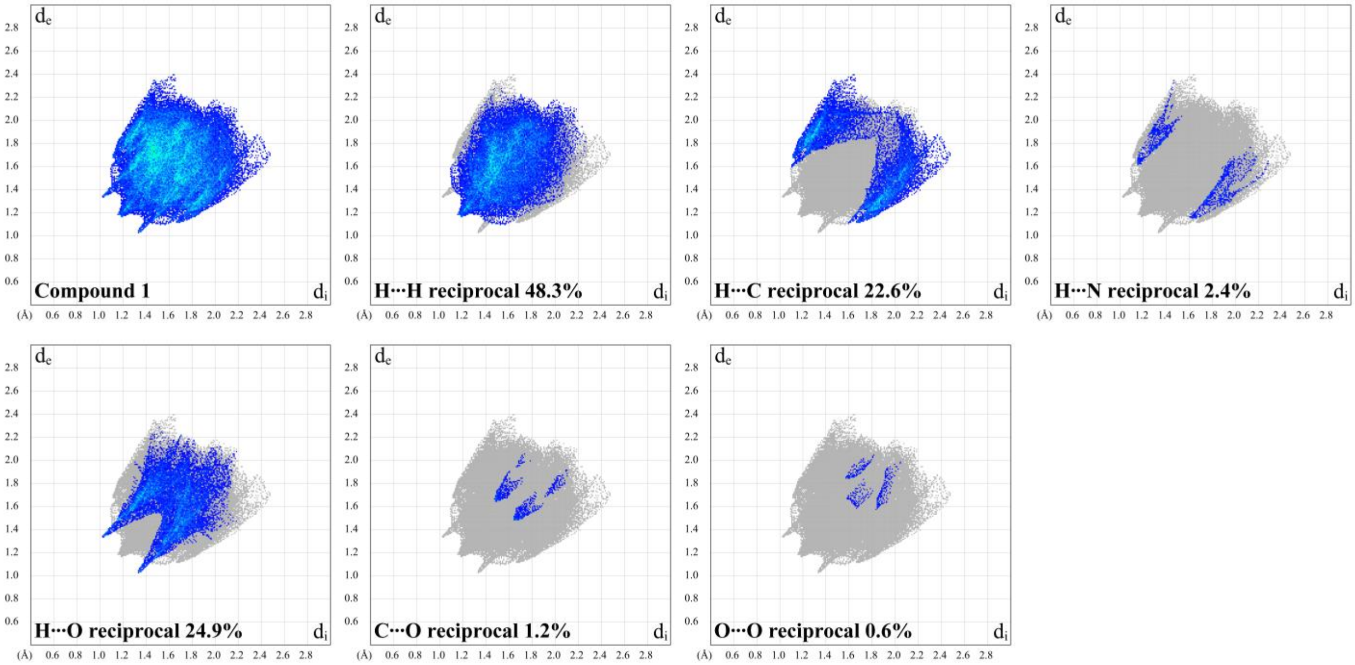


**Figure S1**. 2D and decomposed 2D fingerprint plots of observed contacts for the crystal structure of **1**.

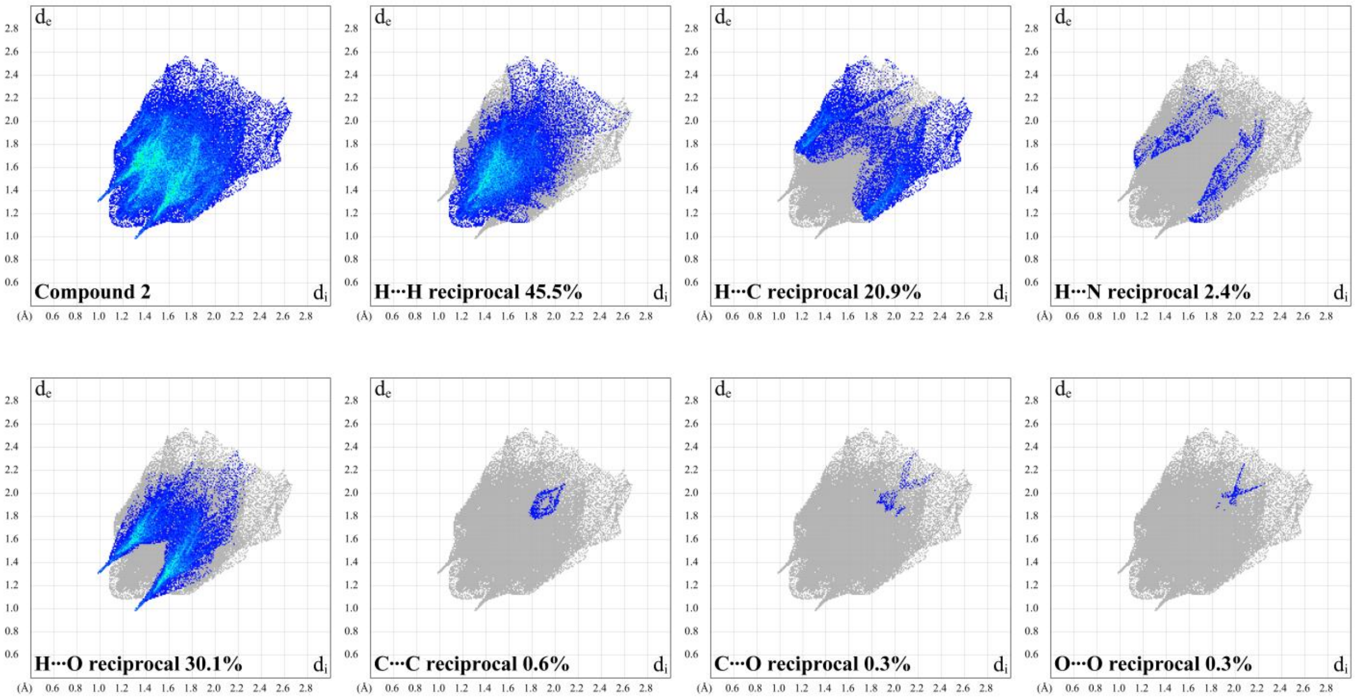


**Figure S2**. 2D and decomposed 2D fingerprint plots of observed contacts for the crystal structure of **2**.

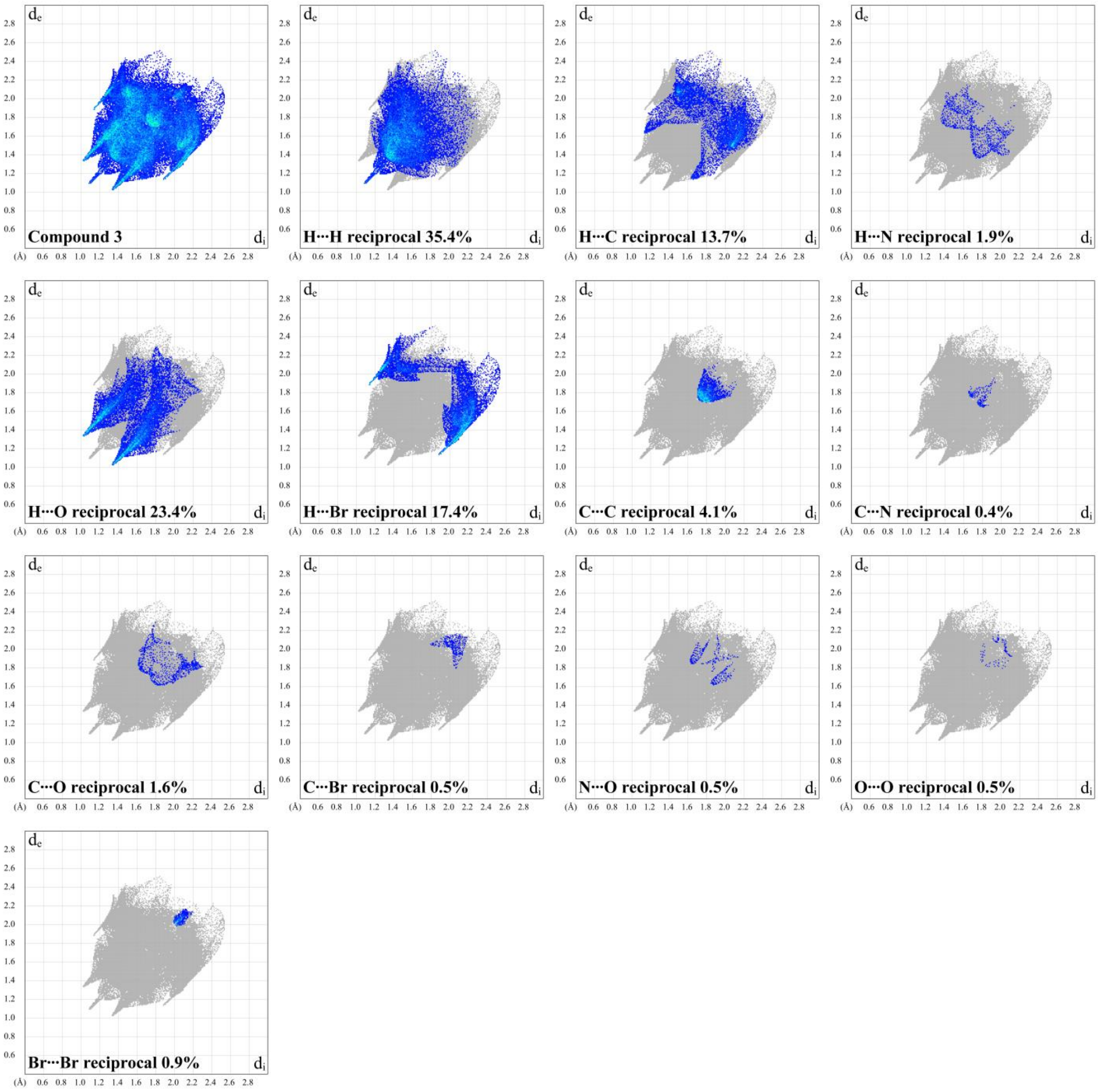


**Figure S3**. 2D and decomposed 2D fingerprint plots of observed contacts for the crystal structure of **3**.

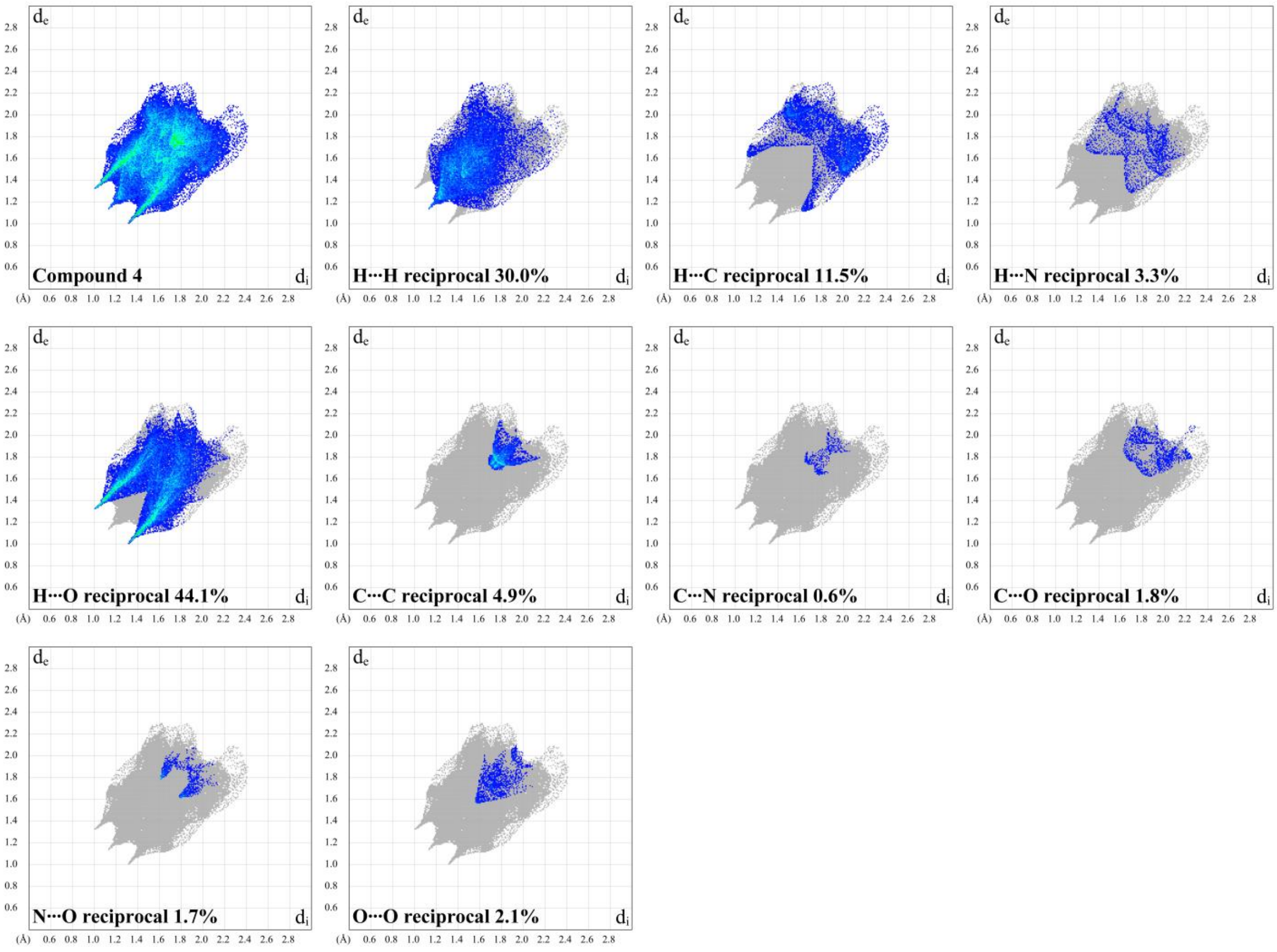


**Figure S4**. 2D and decomposed 2D fingerprint plots of observed contacts for the crystal structure of **4**.

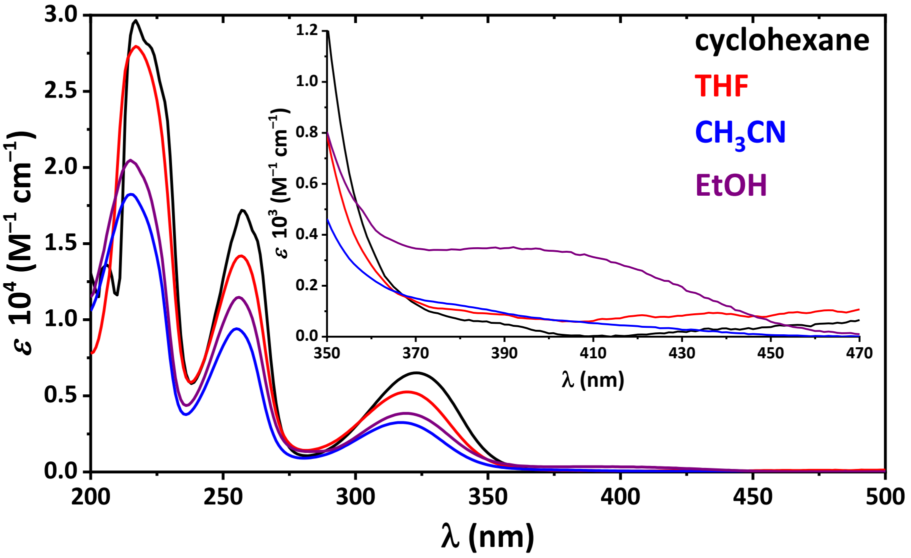


**Figure S5**. UV-vis spectra of **1** in the applied solvents.

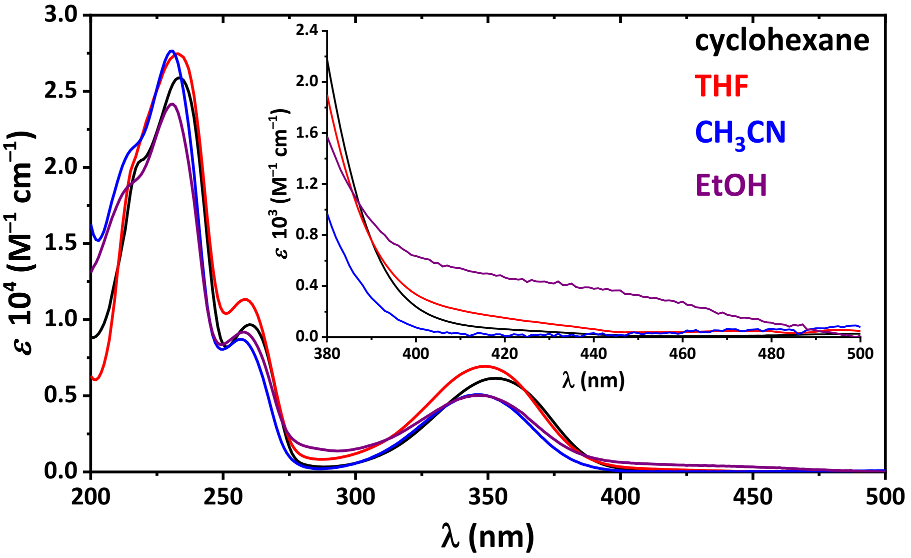


**Figure S6**. UV-vis spectra of **2** in the applied solvents.

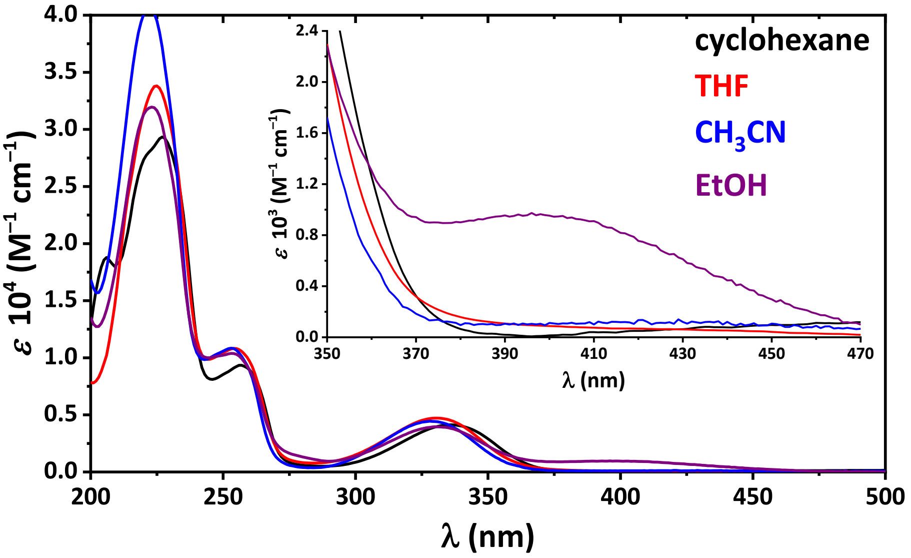


**Figure S7**. UV-vis spectra of **3** in the applied solvents.

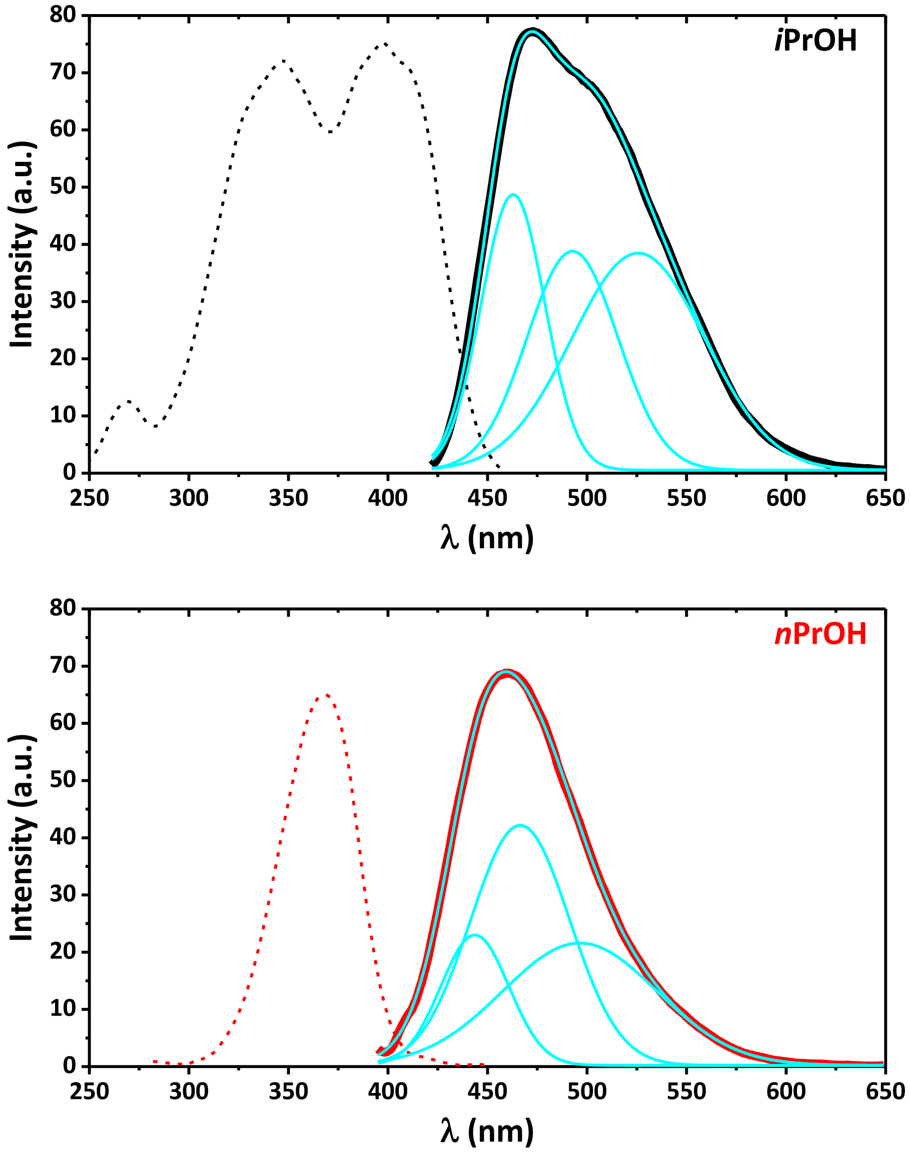


**Figure S8**. Emission (solid line) and excitation (dashed line) spectra of **4** in *n*PrOH ($\lambda_{exc}$ = 360 nm, $\lambda_{em}$ = 460 nm) and *i*PrOH ($\lambda_{exc}$ = 400 nm, $\lambda_{em}$ = 475 nm).

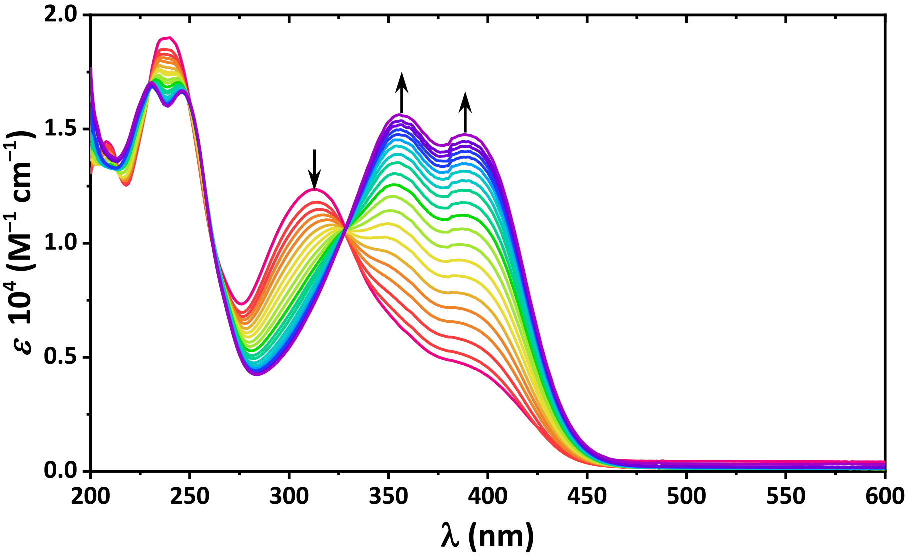


**Figure S9**. UV-vis spectra of **4** in EtOH upon gradual addition of $NEt_3$ up to 2 eqv. with a step of 0.1 eqv.

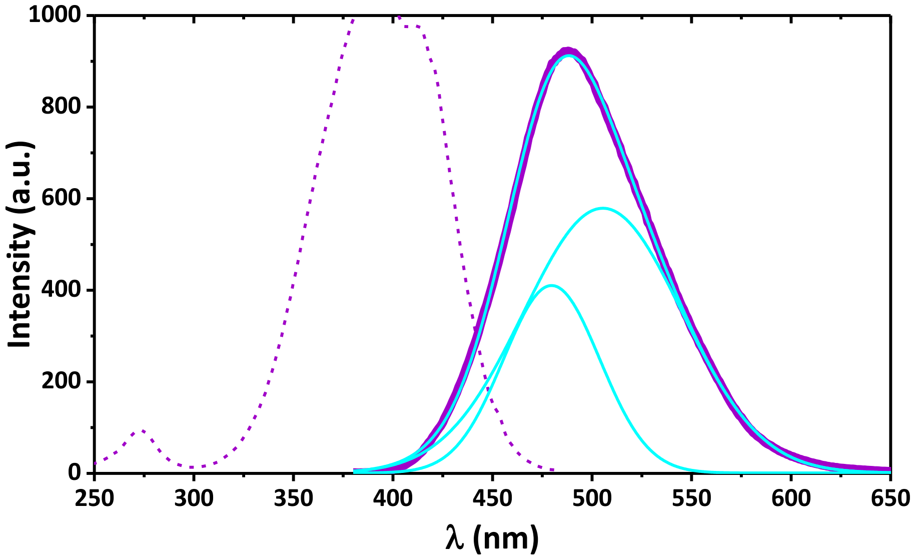


**Figure S10**. Emission (solid line) and excitation (dashed line) spectra of **3** in EtOH after addition of 5 eqv. of NaOH ($\lambda_{exc}$ = 375 nm, $\lambda_{em}$ = 490 nm).

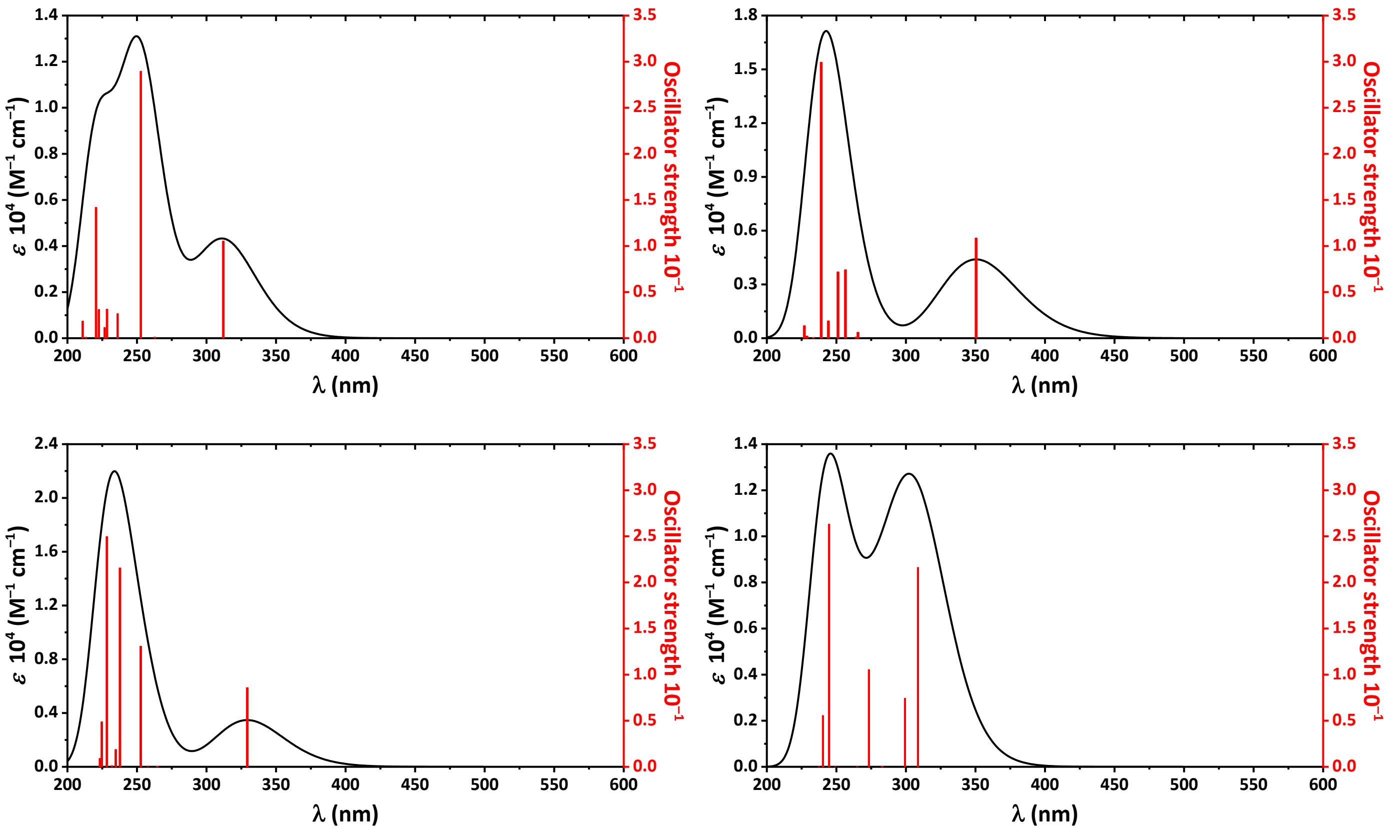


Figure S11. The calculated UV-vis spectra of the ground states of the enol-imine tautomers of **1** (top left), **2** (top right), **3** (bottom left) and **4** (bottom right), obtained by using the TD-DFT/B3LYP/6-311++G(d,p) method.

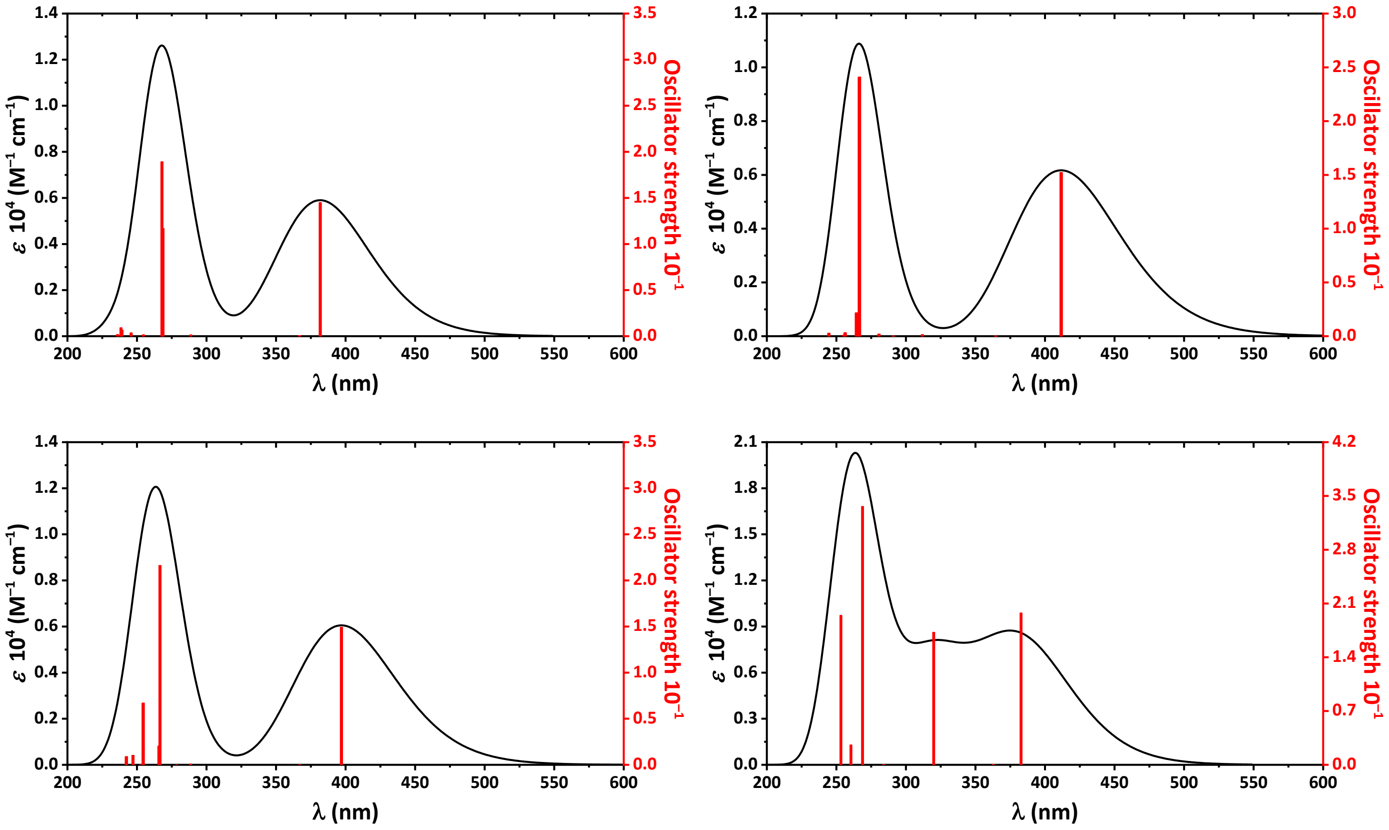


**Figure S12**. The calculated UV-vis spectra of the ground states of the *cis*-keto-enamine tautomers of **1** (top left), **2** (top right), **3** (bottom left) and **4** (bottom right), obtained by using the TD-DFT/B3LYP/6-311++G(d,p) method.

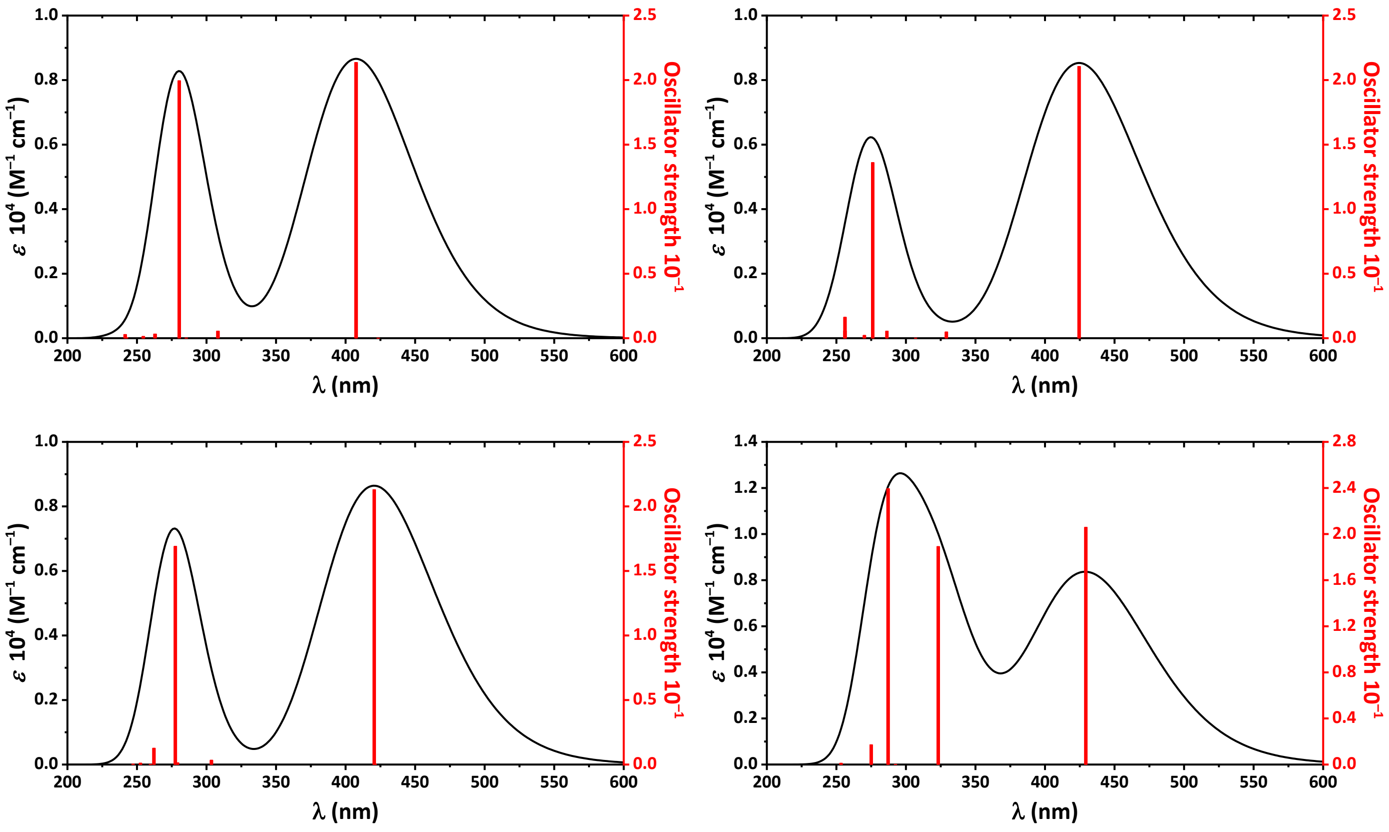


**Figure S13**. The calculated UV-vis spectra of the ground states of the *trans*-keto-enamine tautomers of **1** (top left), **2** (top right), **3** (bottom left) and **4** (bottom right), obtained by using the TD-DFT/B3LYP/6-311++G(d,p) method.

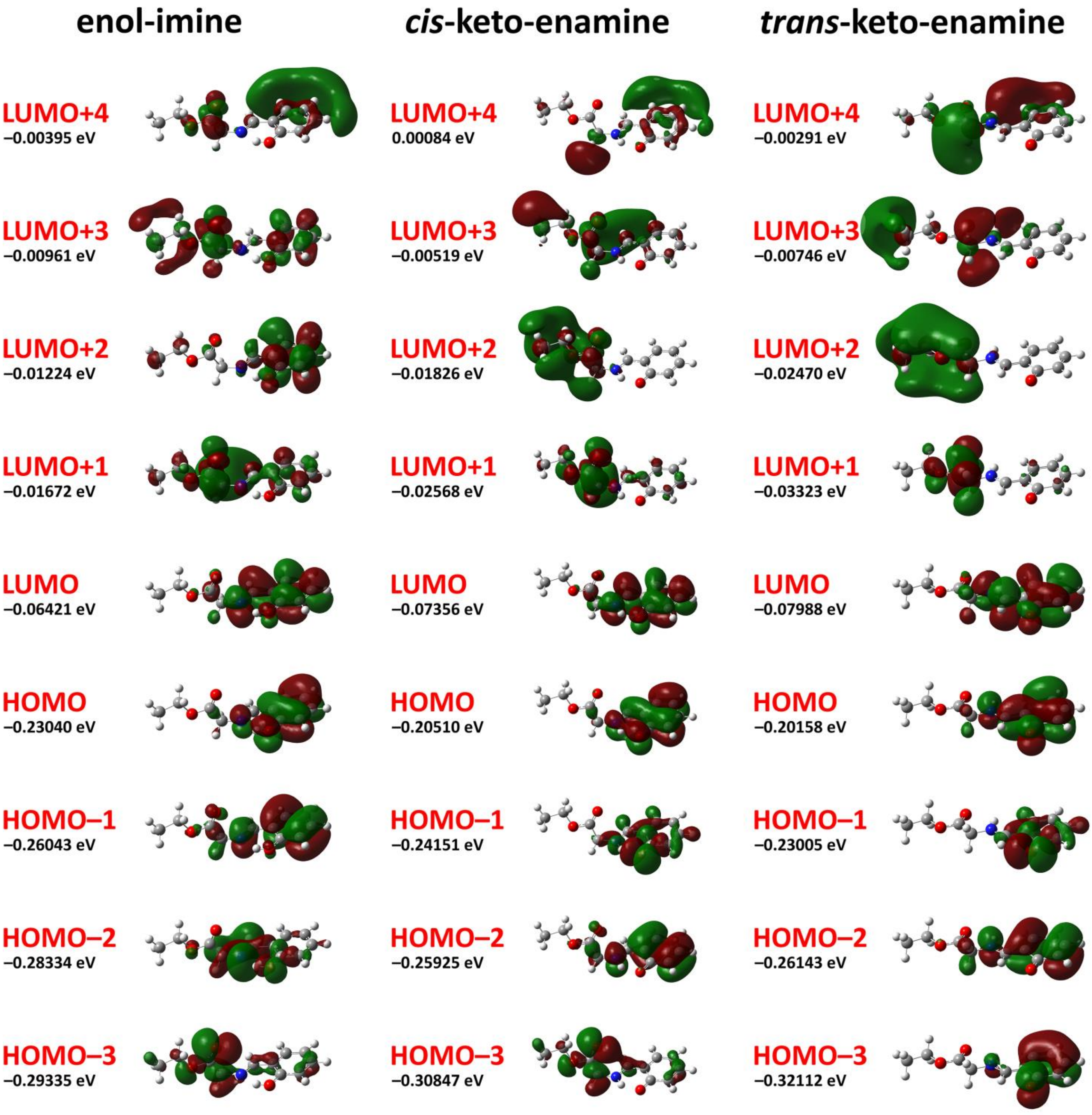


**Figure S14**. Energy levels and views on the electronic isosurfaces of the selected molecular orbitals of the ground state of **1**, obtained by using the B3LYP/6-311++G(d,p) method.

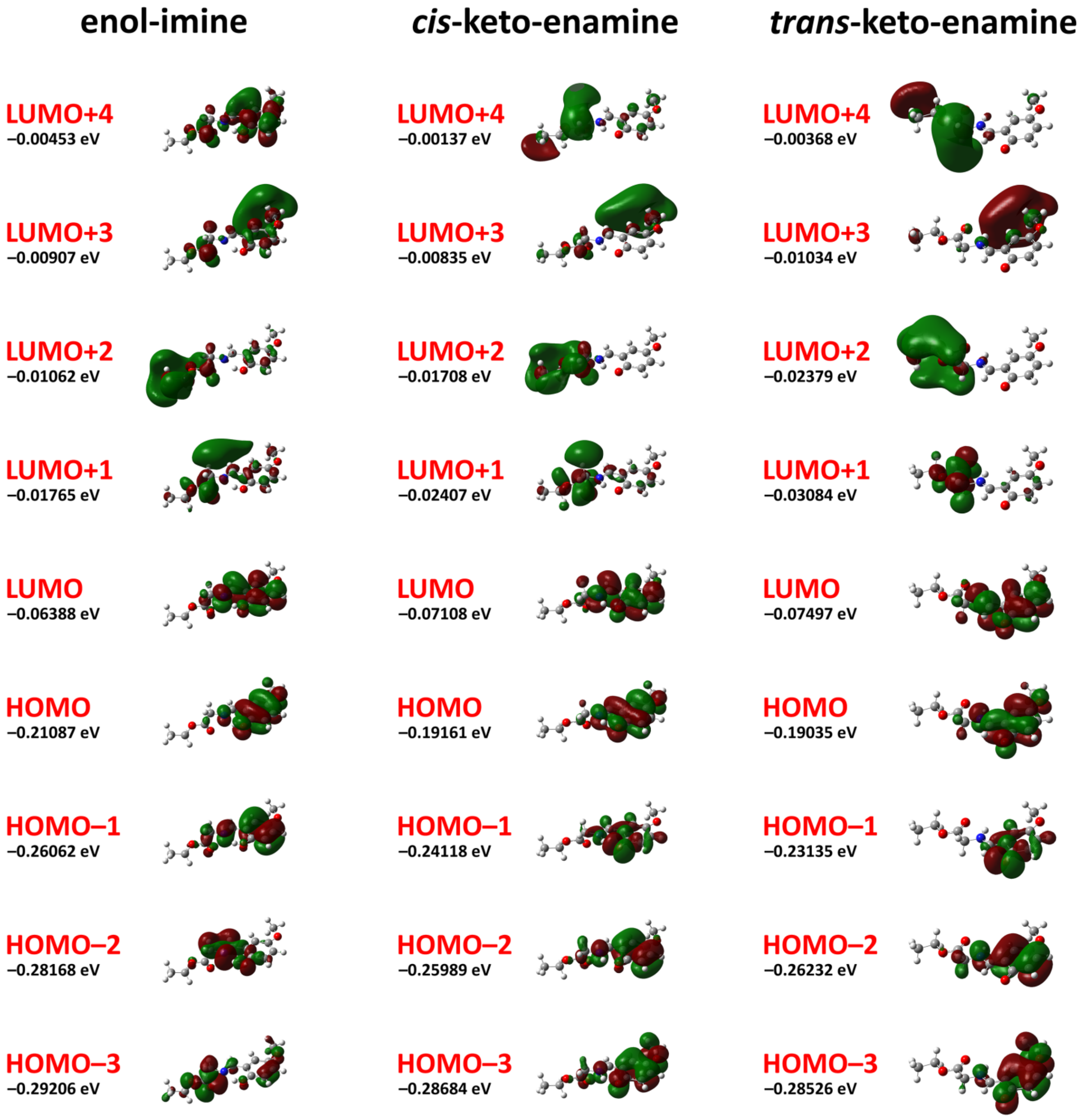


**Figure S15**. Energy levels and views on the electronic isosurfaces of the selected molecular orbitals of the ground state of **2**, obtained by using the B3LYP/6-311++G(d,p) method.

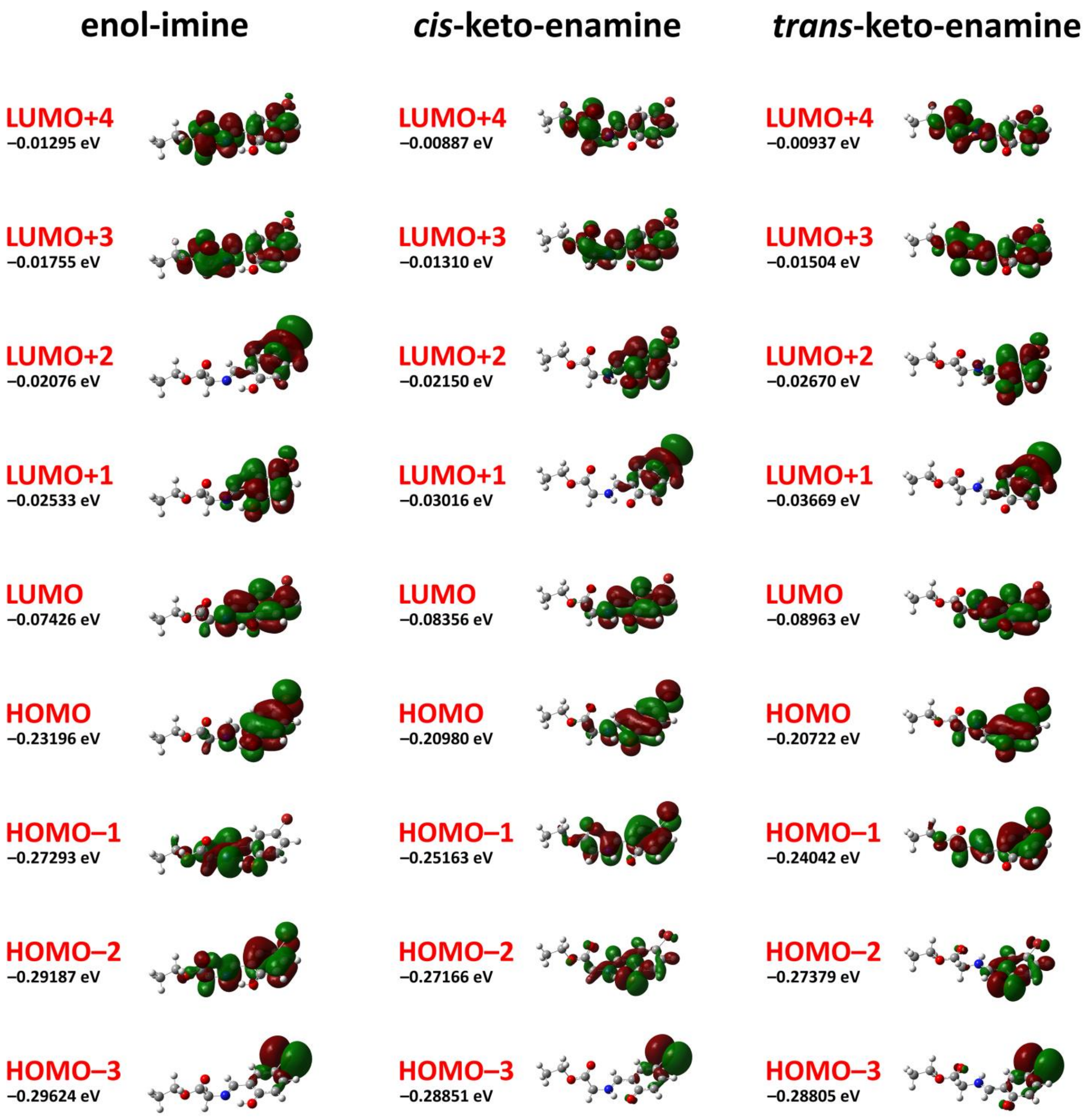


**Figure S16**. Energy levels and views on the electronic isosurfaces of the selected molecular orbitals of the ground state of **3**, obtained by using the B3LYP/6-311++G(d,p) method.

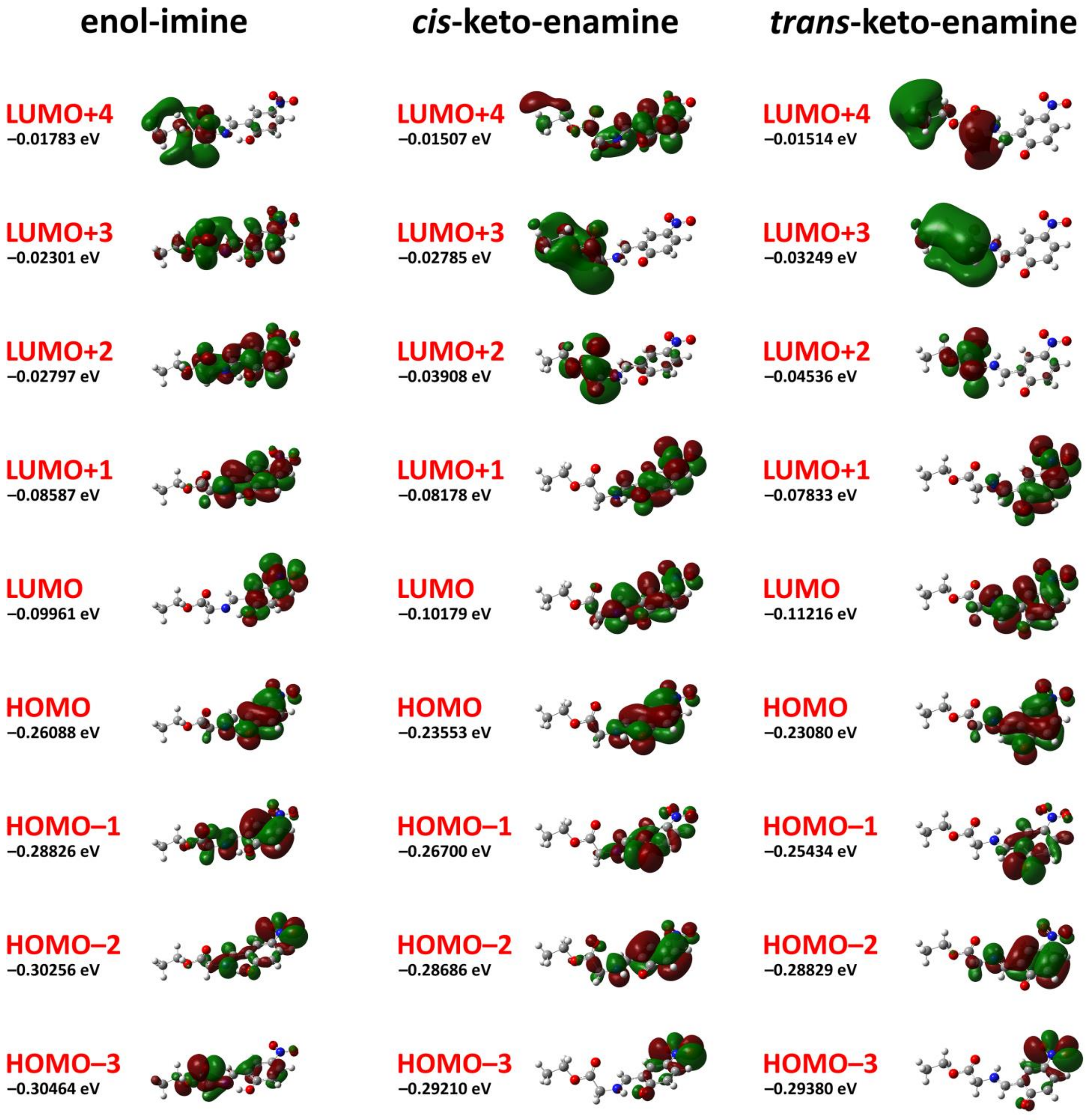


**Figure S17**. Energy levels and views on the electronic isosurfaces of the selected molecular orbitals of the ground state of **4**, obtained by using the B3LYP/6-311++G(d,p) method.

**Table S1**. Values for the main maxima in the experimental UV-vis spectra of **1–4** in different solvents, and in the calculated UV-vis spectra of the ground state for different tautomers of **1–4**, obtained by using the TD-DFT/B3LYP/6-311++G(d,p) method.

| | **Experimental $\lambda_{max}$ (nm)** | | | | **Calculated $\lambda_{max}$ (nm)** | | |
|---|---|---|---|---|---|---|---|
| | cyclohexane | THF | $CH_3CN$ | EtOH | enol-imine | *cis*-keto-enamine | *trans*-keto-enaimne |
| **1** | 217, 257, 323 | 217, 257, 320 | 215, 256, 319 | 215, 255, 317, 392 | 225, 250, 311 | 268, 382 | 280, 407 |
| **2** | 233, 260, 353 | 233, 258, 349 | 231, 257, 346 | 231, 258, 347, 418 | 243, 351 | 266, 412 | 274, 424 |
| **3** | 227, 257, 336 | 225, 254, 331 | 222, 253, 328 | 223, 253, 330, 396 | 234, 329 | 263, 397 | 277, 420 |
| **4** | 217, 236, 255, 307, 351 | 217, 237, 316, 397 | 217, 238, 318, 404 | 209, 240, 313, 389 | 245, 302 | 264, 323, 375 | 295, 428 |

**Table S2**. Values for the calculated UV-vis spectra of the ground state for the *cis*-keto-enamine tautomers of **1–4**, obtained by using the TD-DFT/B3LYP/6-311++G(d,p) method.

| **1** | | | **2** | | |
|---|---|---|---|---|---|
| $\lambda_{max}$ (nm) | Oscillator strength | Transitions | $\lambda_{max}$ (nm) | Oscillator strength | Transitions |
| 238.5 | 0.0092 | HOMO → LUMO+3 (2.9%) | 244.6 | 0.0028 | HOMO → LUMO+5 (13.7%) |
| | | HOMO → LUMO+5 (35.8%) | | | HOMO → LUMO+7 (3.2%) |
| | | HOMO → LUMO+6 (46.4%) | | | HOMO → LUMO+8 (68.8%) |
| | | HOMO → LUMO+8 (8.8%) | | | HOMO → LUMO+9 (12.6%) |
| 239.2 | 0.0068 | HOMO → LUMO+4 (5.5%) | 256.3 | 0.0033 | HOMO–3 → LUMO (4.8%) |
| | | HOMO → LUMO+5 (47.2%) | | | HOMO → LUMO+4 (9.6%) |
| | | HOMO → LUMO+6 (35.5%) | | | HOMO → LUMO+6 (76.9%) |
| | | HOMO → LUMO+8 (3.4%) | | | |
| 267.9 | 0.1893 | HOMO–2 → LUMO (55.6%) | 264.4 | 0.0218 | HOMO–2 → LUMO (9.5%) |
| | | HOMO → LUMO+2 (32.9%) | | | HOMO → LUMO+2 (3.7%) |
| | | HOMO → LUMO+3 (4.1%) | | | HOMO → LUMO+3 (3.0%) |
| | | | | | HOMO → LUMO+4 (72.0%) |
| | | | | | HOMO → LUMO+6 (7.6%) |
| 268.6 | 0.1169 | HOMO–2 → LUMO (35.3%) | 266.6 | 0.2410 | HOMO–2 → LUMO (79.4%) |
| | | HOMO → LUMO+2 (52.5%) | | | HOMO → LUMO+4 (6.0%) |
| | | HOMO → LUMO+3 (7.5%) | | | HOMO → LUMO+6 (3.2%) |
| | | | | | HOMO → LUMO+14 (4.5%) |
| 381.8 | 0.1450 | HOMO → LUMO (97.6%) | 411.6 | 0.1523 | HOMO → LUMO (98.8%) |

| **3** | | | **4** | | |
|---|---|---|---|---|---|
| $\lambda_{max}$ (nm) | Oscillator strength | Transitions | $\lambda_{max}$ (nm) | Oscillator strength | Transitions |
| 247.1 | 0.0103 | HOMO–3 → LUMO (38.1%) | 253.2 | 0.1945 | HOMO–3 → LUMO (7.2%) |
| | | HOMO → LUMO+2 (4.2%) | | | HOMO–3 → LUMO+1 (83.5%) |
| | | HOMO → LUMO+3 (2.2%) | | | |
| | | HOMO → LUMO+4 (46.5%) | | | |
| | | HOMO → LUMO+6 (2.1%) | | | |
| 254.5 | 0.0670 | HOMO–3 → LUMO (46.7%) | 260.4 | 0.0259 | HOMO–2 → LUMO (2.7%) |
| | | HOMO → LUMO+4 (34.2%) | | | HOMO → LUMO+2 (94.5%) |
| | | HOMO → LUMO+5 (13.5%) | | | |
| 265.8 | 0.0204 | HOMO–2 → LUMO (6.6%) | 268.8 | 0.3364 | HOMO–2 → LUMO (80.4%) |
| | | HOMO → LUMO+2 (81.5%) | | | HOMO–2 → LUMO+1 (8.8%) |
| | | HOMO → LUMO+4 (6.0%) | | | HOMO → LUMO+1 (2.6%) |
| | | HOMO → LUMO+5 (2.8%) | | | HOMO → LUMO+2 (2.1%) |
| 266.6 | 0.2162 | HOMO–2 → LUMO (81.6%) | 320.0 | 0.1726 | HOMO–2 → LUMO (2.7%) |
| | | HOMO → LUMO+2 (6.2%) | | | HOMO → LUMO+1 (95.3%) |
| | | HOMO → LUMO+4 (2.3%) | | | |
| | | HOMO → LUMO+10 (5.6%) | | | |
| 397.1 | 0.1493 | HOMO → LUMO (98.6%) | 382.8 | 0.1977 | HOMO → LUMO (97.7%) |

**Table S3**. Values for the calculated UV-vis spectra of the ground state for the *trans*-keto-enamine tautomers of **1–4**, obtained by using the TD-DFT/B3LYP/6-311++G(d,p) method.

| **1** | | | **2** | | |
|---|---|---|---|---|---|
| $\lambda_{max}$ (nm) | Oscillator strength | Transitions | $\lambda_{max}$ (nm) | Oscillator strength | Transitions |
| 241.5 | 0.0029 | HOMO–3 → LUMO (2.2%) | 256.2 | 0.0058 | HOMO → LUMO+5 (54.9%) |
| | | HOMO → LUMO+5 (80.3%) | | | HOMO → LUMO+6 (19.5%) |
| | | HOMO → LUMO+7 (14.7%) | | | HOMO → LUMO+7 (16.4%) |
| | | | | | HOMO → LUMO+8 (3.1%) |
| 263.0 | 0.0033 | HOMO → LUMO+2 (6.1%) | 256.2 | 0.0163 | HOMO–3 → LUMO (2.8%) |
| | | HOMO → LUMO+3 (40.8%) | | | HOMO–2 → LUMO (2.7%) |
| | | HOMO → LUMO+4 (45.3%) | | | HOMO → LUMO+5 (18.2%) |
| | | HOMO → LUMO+6(6.2%) | | | HOMO → LUMO+6 (58.8%) |
| | | | | | HOMO → LUMO+7 (5.6%) |
| | | | | | HOMO → LUMO+8 (9.1%) |
| 280.4 | 0.1995 | HOMO–2 → LUMO (92.7%) | 276.1 | 0.1361 | HOMO–3 → LUMO (2.3%) |
| | | HOMO → LUMO+10 (3.3%) | | | HOMO–2 → LUMO (87.3%) |
| | | | | | HOMO → LUMO+8 (3.4%) |
| 308.2 | 0.0056 | HOMO → LUMO+1 (99.2%) | 329.1 | 0.0049 | HOMO → LUMO+1 (99.4%) |
| 407.5 | 0.2137 | HOMO → LUMO (98.9%) | 424.5 | 0.2106 | HOMO → LUMO (99.6%) |
| | | | | | HOMO ← LUMO (2.1%) |
| **3** | | | **4** | | |
| $\lambda_{max}$ (nm) | Oscillator strength | Transitions | $\lambda_{max}$ (nm) | Oscillator strength | Transitions |
| 262.2 | 0.0127 | HOMO–3 → LUMO (85.3%) | 253.4 | 0.0012 | HOMO → LUMO+3 (91.0%) |
| | | HOMO → LUMO+6 (8.4%) | | | HOMO → LUMO+4 (7.5%) |
| | | HOMO → LUMO+7 (4.1%) | | | |
| 277.6 | 0.1691 | HOMO–2 → LUMO (90.9%) | 275.0 | 0.0172 | HOMO → LUMO+2 (97.1%) |
| | | HOMO → LUMO+10 (5.4%) | | | |
| 279.2 | 0.0014 | HOMO → LUMO+2 (85.9%) | 287.2 | 0.2395 | HOMO–2 → LUMO (89.4%) |
| | | HOMO → LUMO+3 (3.8%) | | | HOMO → LUMO+1 (2.3%) |
| | | HOMO → LUMO+4 (9.0%) | | | HOMO → LUMO+2 (2.1%) |
| 303.5 | 0.0034 | HOMO → LUMO+1 (99.2%) | 323.2 | 0.1892 | HOMO–2 → LUMO (2.4%) |
| | | | | | HOMO → LUMO+1 (95.4%) |
| 420.6 | 0.2130 | HOMO → LUMO (99.3%) | 429.4 | 0.2059 | HOMO → LUMO (98.6%) |

**Table S4.** Cartesian atomic coordinates for the optimized structures of **1**, obtained by using the DFT/B3LYP/6–311++G(d,p) method.

| enol-imine tautomer | | | | *cis*-keto-enamine tautomer | | | | *trans*-keto-enamine tautomer | | | |
|---|---|---|---|---|---|---|---|---|---|---|---|
| Atom | *x* | *y* | *z* | Atom | *x* | *y* | *z* | Atom | *x* | *y* | *z* |
| C1 | 3.909396 | 1.198362 | –0.284570 | C1 | 3.981774 | 0.949362 | –0.623489 | C1 | 4.682733 | 0.410773 | 0.000266 |
| C2 | 2.582566 | 0.911321 | 0.060196 | C2 | 2.605675 | 0.945330 | –0.192517 | C2 | 3.363638 | 1.039599 | –0.000075 |
| C3 | 2.176368 | –0.441352 | 0.200683 | C3 | 2.094958 | –0.335779 | 0.304993 | C3 | 2.215010 | 0.094719 | –0.000054 |
| C4 | 3.119888 | –1.461892 | –0.012678 | C4 | 2.940319 | –1.484665 | 0.345542 | C4 | 2.444855 | –1.322040 | –0.000006 |
| C5 | 4.429927 | –1.171073 | –0.354208 | C5 | 4.238918 | –1.419191 | –0.074729 | C5 | 3.706285 | –1.831199 | –0.000074 |
| C6 | 4.816417 | 0.168955 | –0.488022 | C6 | 4.750267 | –0.180559 | –0.562312 | C6 | 4.833370 | –0.939677 | 0.000001 |
| H7 | 4.200241 | 2.236738 | –0.387751 | H7 | 4.377526 | 1.886799 | –0.996346 | H7 | 5.535531 | 1.079739 | 0.000811 |
| H8 | 2.801043 | –2.494282 | 0.093019 | H8 | 2.528469 | –2.419478 | 0.715723 | H8 | 1.599624 | –2.005122 | 0.000226 |
| H9 | 5.145456 | –1.967546 | –0.517088 | H9 | 4.877421 | –2.293362 | –0.044172 | H9 | 3.870874 | –2.901892 | 0.000190 |
| H10 | 5.839513 | 0.409438 | –0.755743 | H10 | 5.782879 | –0.138163 | –0.895403 | H10 | 5.831587 | –1.367363 | 0.000614 |
| O11 | 1.732175 | 1.930610 | 0.250156 | O11 | 1.882005 | 1.979527 | –0.236728 | O11 | 3.220815 | 2.269251 | –0.000319 |
| H12 | 0.845257 | 1.544671 | 0.473692 | C12 | 0.767894 | –0.430547 | 0.731637 | C12 | 0.962383 | 0.673786 | 0.000037 |
| C13 | 0.809711 | –0.783498 | 0.554377 | H13 | 0.381052 | –1.382302 | 1.088863 | N13 | –0.227733 | 0.059690 | –0.000236 |
| H14 | 0.581679 | –1.855079 | 0.636066 | N14 | –0.062441 | 0.602075 | 0.732853 | C14 | –1.492461 | 0.764786 | 0.000199 |
| N15 | –0.093198 | 0.100551 | 0.768076 | C15 | –1.454562 | 0.534380 | 1.100500 | H15 | –1.599284 | 1.405861 | 0.882381 |
| C16 | –1.433182 | –0.330052 | 1.093510 | H16 | –1.831444 | 1.542630 | 1.269667 | H16 | –1.599211 | 1.407088 | –0.880989 |
| H17 | –1.827595 | 0.271687 | 1.914389 | H17 | –1.582745 | –0.015324 | 2.040688 | C17 | –2.629847 | –0.241886 | –0.000122 |
| H18 | –1.472028 | –1.38535 | 1.407309 | C18 | –2.326870 | –0.164612 | 0.052879 | O18 | –2.469530 | –1.439254 | –0.000720 |
| C19 | –2.374614 | –0.201486 | –0.103166 | O19 | –1.923543 | –0.947817 | –0.766806 | O19 | –3.811366 | 0.376711 | 0.000518 |
| O20 | –2.043943 | –0.284450 | –1.257249 | O20 | –3.610270 | 0.189646 | 0.214440 | C20 | –4.999183 | –0.469649 | 0.000922 |
| O21 | –3.642034 | –0.029178 | 0.316622 | C21 | –4.579747 | –0.448561 | –0.665476 | H21 | –4.961828 | –1.107926 | 0.885771 |
| C22 | –4.664091 | 0.024755 | –0.714557 | H22 | –4.295960 | –0.234882 | –1.697709 | H22 | –4.961027 | –1.109977 | –0.882382 |
| H23 | –4.423553 | 0.843958 | –1.395213 | H23 | –4.519661 | –1.528587 | –0.515133 | C23 | –6.207350 | 0.441426 | –0.000646 |
| H24 | –4.629527 | –0.906423 | –1.284724 | C24 | –5.945504 | 0.104286 | –0.319392 | H24 | –7.117871 | –0.163250 | –0.000434 |
| C25 | –5.998477 | 0.225049 | –0.027416 | H25 | –5.980847 | 1.185453 | –0.469186 | H25 | –6.219996 | 1.079523 | 0.885420 |
| H26 | –6.011165 | 1.158004 | 0.540278 | H26 | –6.698718 | –0.355478 | –0.964554 | H26 | –6.219133 | 1.077559 | –0.888144 |
| H27 | –6.793032 | 0.269195 | –0.777015 | H27 | –6.206520 | –0.111639 | 0.718936 | H27 | –0.301080 | –0.950557 | –0.000676 |
| H28 | –6.216967 | –0.599250 | 0.655122 | H28 | 0.381363 | 1.462296 | 0.345584 | H28 | 0.939993 | 1.761002 | 0.000346 |

**Table S5.** Cartesian atomic coordinates for the optimized structures of **2**, obtained by using the DFT/B3LYP/6–311++G(d,p) method.

| Enol-imine tautomer | | | | *Cis*-keto-enamine tautomer | | | | *Trans*-keto-enamine tautomer | | | |
|---|---|---|---|---|---|---|---|---|---|---|---|
| Atom | *x* | *y* | *z* | Atom | *x* | *y* | *z* | Atom | *x* | *y* | *z* |
| C1 | –2.989397 | 1.990841 | –0.370350 | C1 | –3.110512 | 1.896766 | –0.575500 | C1 | –3.415254 | 0.902318 | 0.000118 |
| C2 | –1.704654 | 1.563907 | –0.003463 | C2 | –1.749093 | 1.661443 | –0.148794 | C2 | –2.094066 | 0.574314 | 0.000040 |
| C3 | –1.496217 | 0.197373 | 0.285550 | C3 | –1.446651 | 0.297305 | 0.280940 | C3 | –1.687681 | –0.810127 | –0.000035 |
| C4 | –2.576175 | –0.709830 | 0.198120 | C4 | –2.459542 | –0.720852 | 0.269516 | C4 | –2.697840 | –1.895336 | 0.000167 |
| C5 | –3.838165 | –0.271998 | –0.168258 | C5 | –3.726691 | –0.426054 | –0.145919 | C5 | –4.091451 | –1.443757 | 0.000146 |
| C6 | –4.034434 | 1.090855 | –0.451231 | C6 | –4.041611 | 0.902802 | –0.570203 | C6 | –4.419917 | –0.131086 | 0.000093 |
| H7 | –3.140552 | 3.040624 | –0.590444 | H7 | –3.358985 | 2.900106 | –0.900417 | H7 | –1.339556 | 1.351584 | 0.000204 |
| H8 | –2.387017 | –1.752357 | 0.421321 | H8 | –2.186736 | –1.718378 | 0.592390 | H8 | –4.850482 | –2.217173 | 0.000166 |
| H9 | –5.027065 | 1.419084 | –0.736265 | H9 | –5.060025 | 1.092230 | –0.892730 | H9 | –5.456339 | 0.190163 | 0.000025 |
| O10 | –0.717079 | 2.474326 | 0.062081 | O10 | –0.883028 | 2.579386 | –0.147276 | C10 | –0.373448 | –1.218242 | –0.000371 |
| H11 | 0.111574 | 1.996114 | 0.321422 | C11 | –0.154918 | –0.017064 | 0.698704 | H11 | –0.203096 | –2.291346 | –0.000259 |
| C12 | –0.186200 | –0.296125 | 0.671371 | H12 | 0.081734 | –1.032240 | 1.008667 | C12 | 2.072913 | –0.970822 | –0.000621 |
| H13 | –0.111757 | –1.372460 | 0.879566 | N13 | 0.831434 | 0.873763 | 0.751375 | H13 | 2.268187 | –1.592349 | –0.882254 |
| N14 | 0.839546 | 0.465894 | 0.776354 | C14 | 2.191399 | 0.564612 | 1.109726 | H14 | 2.267463 | –1.593128 | 0.880608 |
| C15 | 2.109277 | –0.116399 | 1.144559 | H15 | 2.226786 | –0.046919 | 2.019700 | C15 | 3.065693 | 0.178170 | 0.000220 |
| H16 | 1.998917 | –1.112543 | 1.602313 | H16 | 2.724283 | 1.488918 | 1.332184 | O16 | 4.320069 | –0.277090 | 0.000797 |
| H17 | 2.616023 | 0.524207 | 1.868340 | C17 | 2.952158 | –0.206453 | 0.026153 | C17 | 5.383349 | 0.720141 | 0.000447 |
| C18 | 3.019766 | –0.301404 | –0.068831 | O18 | 2.440491 | –0.854083 | –0.849467 | H18 | 5.261550 | 1.348761 | 0.884661 |
| O19 | 2.642272 | –0.526069 | –1.189358 | O19 | 4.274605 | –0.090202 | 0.224683 | H19 | 5.260579 | 1.349141 | –0.883381 |
| O20 | 4.312330 | –0.226994 | 0.299523 | C20 | 5.139244 | –0.825877 | –0.686283 | C20 | 6.702972 | –0.020612 | –0.000370 |
| C21 | 5.297268 | –0.468213 | –0.740909 | H21 | 4.882433 | –1.885525 | –0.623354 | H21 | 7.524193 | 0.700738 | –0.000508 |
| H22 | 5.127567 | –1.465430 | –1.153348 | H22 | 4.930540 | –0.487871 | –1.703183 | H22 | 6.800906 | –0.650590 | 0.886190 |
| H23 | 5.137527 | 0.257338 | –1.541118 | C23 | 6.571187 | –0.557513 | –0.275234 | H23 | 6.800131 | –0.650096 | –0.887360 |
| C24 | 6.668866 | –0.334701 | –0.113333 | H24 | 6.756685 | –0.894436 | 0.746986 | O24 | 2.749701 | 1.344434 | 0.000271 |
| H25 | 6.806062 | –1.061816 | 0.690141 | H25 | 7.249213 | –1.096561 | –0.942073 | O25 | –3.934513 | 2.167663 | 0.000256 |
| H26 | 7.436874 | –0.511879 | –0.870937 | H26 | 6.804698 | 0.507477 | –0.337898 | C26 | –3.026041 | 3.256838 | –0.000310 |
| H27 | 6.816803 | 0.666825 | 0.296425 | O27 | –4.781265 | –1.301312 | –0.207789 | H27 | –2.391779 | 3.244804 | –0.894362 |
| O28 | –4.947591 | –1.067850 | –0.283774 | C28 | –4.548058 | –2.642391 | 0.186372 | H28 | –3.635555 | 4.159039 | –0.000640 |
| C29 | –4.805358 | –2.455041 | –0.024231 | H29 | –5.495200 | –3.164554 | 0.058973 | H29 | –2.391728 | 3.245628 | 0.893725 |
| H30 | –5.791378 | –2.891346 | –0.176127 | H30 | –3.784519 | –3.116208 | –0.441952 | N30 | 0.726270 | –0.443811 | –0.000914 |
| H31 | –4.093336 | –2.921287 | –0.715150 | H31 | –4.239223 | –2.702369 | 1.236944 | H31 | 0.661108 | 0.565906 | –0.000735 |
| H32 | –4.483995 | –2.639225 | 1.007632 | H32 | 0.540391 | 1.809335 | 0.408055 | O32 | –2.407114 | –3.098156 | 0.000324 |

**Table S6.** Cartesian atomic coordinates for the optimized structures of **3**, obtained by using the DFT/B3LYP/6–311++G(d,p) method.

| Enol-imine tautomer | | | | *Cis*-keto-enamine tautomer | | | | *Trans*-keto-enamine tautomer | | | |
|---|---|---|---|---|---|---|---|---|---|---|---|
| Atom | *x* | *y* | *z* | Atom | *x* | *y* | *z* | Atom | *x* | *y* | *z* |
| C1 | 2.163472 | 2.237242 | −0.288396 | C1 | 2.299267 | 2.224618 | −0.614265 | C1 | 3.277298 | 2.202277 | 0.000075 |
| C2 | 0.903433 | 1.719169 | 0.037718 | C2 | 0.947509 | 1.930585 | −0.201754 | C2 | 1.840245 | 2.473959 | 0.000141 |
| C3 | 0.776869 | 0.335775 | 0.322864 | C3 | 0.713650 | 0.572264 | 0.291909 | C3 | 0.973320 | 1.268394 | −0.000040 |
| C4 | 1.916201 | −0.486263 | 0.270501 | C4 | 1.777098 | −0.377551 | 0.347911 | C4 | 1.550814 | −0.045706 | −0.000079 |
| C5 | 3.148061 | 0.049159 | −0.053641 | C5 | 3.028667 | −0.023467 | −0.058734 | C5 | 2.899316 | −0.196681 | 0.000001 |
| C6 | 3.277031 | 1.412718 | −0.334505 | C6 | 3.292687 | 1.288773 | −0.543992 | C6 | 3.775924 | 0.939732 | 0.000113 |
| H7 | 2.246735 | 3.295099 | −0.505330 | H7 | 2.497190 | 3.223575 | −0.984153 | H7 | 3.933767 | 3.064489 | −0.000181 |
| H8 | 1.817369 | −1.543614 | 0.486440 | H8 | 1.574513 | −1.378219 | 0.712906 | H8 | 0.915142 | −0.924349 | −0.000315 |
| H9 | 4.245295 | 1.824978 | −0.588747 | H9 | 4.298565 | 1.538883 | −0.860446 | H9 | 4.846585 | 0.771943 | −0.000183 |
| O10 | −0.144810 | 2.551601 | 0.071621 | O10 | 0.025593 | 2.789077 | −0.259640 | O10 | 1.392033 | 3.626332 | 0.000342 |
| H11 | −0.947659 | 2.015724 | 0.305762 | C11 | −0.568524 | 0.196653 | 0.706315 | C11 | −0.387619 | 1.506322 | −0.000224 |
| C12 | −0.510615 | −0.248419 | 0.665555 | H12 | −0.747312 | −0.814332 | 1.063710 | N12 | −1.380192 | 0.610193 | 0.000277 |
| H13 | −0.520603 | −1.329070 | 0.862114 | N13 | −1.598287 | 1.028521 | 0.693140 | C13 | −2.784426 | 0.968748 | −0.000531 |
| N14 | −1.581903 | 0.448005 | 0.741463 | C14 | −2.947763 | 0.667757 | 1.053541 | H14 | −3.050734 | 1.562297 | 0.880777 |
| C15 | −2.825084 | −0.206781 | 1.076248 | H15 | −3.545001 | 1.573320 | 1.154800 | H15 | −3.050380 | 1.560011 | −0.883520 |
| H16 | −3.323979 | 0.336984 | 1.881614 | H16 | −2.971219 | 0.161568 | 2.025888 | C16 | −3.623669 | −0.297882 | 0.000783 |
| H17 | −2.678245 | −1.242858 | 1.419529 | C17 | −3.615101 | −0.270879 | 0.043118 | O17 | −3.156977 | −1.412039 | 0.002146 |
| C18 | −3.769299 | −0.269539 | −0.122710 | O18 | −3.025878 | −0.962856 | −0.745639 | O18 | −4.923986 | −0.006187 | 0.000082 |
| O19 | −3.430706 | −0.236219 | −1.276638 | O19 | −4.945182 | −0.240157 | 0.201007 | C19 | −5.852556 | −1.132345 | 0.000692 |
| O20 | −5.039621 | −0.412282 | 0.297817 | C20 | −5.725480 | −1.137738 | −0.641259 | H20 | −5.650632 | −1.737979 | 0.886113 |
| C21 | −6.050840 | −0.566969 | −0.734693 | H21 | −5.517131 | −0.892674 | −1.684402 | H21 | −5.648436 | −1.740871 | −0.882230 |
| H22 | −6.015326 | 0.309552 | −1.384836 | H22 | −5.385793 | −2.159376 | −0.458954 | C22 | −7.255437 | −0.565627 | −0.001920 |
| H23 | −5.798202 | −1.442503 | −1.336981 | C23 | −7.184008 | −0.945402 | −0.286058 | H23 | −7.977731 | −1.385942 | −0.001489 |
| C24 | −7.390895 | −0.713946 | −0.045330 | H24 | −7.500578 | 0.083667 | −0.468666 | H24 | −7.433644 | 0.047672 | 0.883799 |
| H25 | −7.621661 | 0.168269 | 0.555759 | H25 | −7.798713 | −1.607060 | −0.901858 | H25 | −7.431485 | 0.044852 | −0.890013 |
| H26 | −8.177042 | −0.832399 | −0.795694 | H26 | −7.368396 | −1.185412 | 0.763290 | H26 | −1.195972 | −0.386342 | 0.001514 |
| H27 | −7.404178 | −1.590679 | 0.605943 | Br27 | 4.470142 | −1.289974 | 0.009429 | H27 | −0.685178 | 2.552198 | −0.000784 |
| Br28 | 4.695483 | −1.082949 | −0.119996 | H28 | −1.347352 | 1.963309 | 0.309682 | Br28 | 3.689206 | −1.945466 | −0.000287 |

**Table S7.** Cartesian atomic coordinates for the optimized structures of **4**, obtained by using the DFT/B3LYP/6–311++G(d,p) method.

| Enol-imine tautomer | | | | *Cis*-keto-enamine tautomer | | | | *Trans*-keto-enamine tautomer | | | |
|---|---|---|---|---|---|---|---|---|---|---|---|
| Atom | *x* | *y* | *z* | Atom | *x* | *y* | *z* | Atom | *x* | *y* | *z* |
| C1 | 2.679264 | 2.027798 | –0.185635 | C1 | 2.838151 | 1.962604 | –0.570747 | C1 | 3.771804 | 1.738400 | –0.000047 |
| C2 | 1.401628 | 1.525894 | 0.114928 | C2 | 1.469966 | 1.740071 | –0.160324 | C2 | 2.359491 | 2.118085 | 0.000006 |
| C3 | 1.225730 | 0.127124 | 0.316921 | C3 | 1.150003 | 0.383116 | 0.304141 | C3 | 1.398028 | 0.980399 | 0.000024 |
| C4 | 2.330295 | –0.721228 | 0.209922 | C4 | 2.141908 | –0.621229 | 0.338975 | C4 | 1.864052 | –0.359168 | 0.000069 |
| C5 | 3.578379 | –0.198263 | –0.086921 | C5 | 3.420232 | –0.331319 | –0.063368 | C5 | 3.205268 | –0.615574 | 0.000010 |
| C6 | 3.760725 | 1.175265 | –0.286431 | C6 | 3.771246 | 0.970112 | –0.523220 | C6 | 4.172360 | 0.443210 | –0.000044 |
| H7 | 2.790236 | 3.094080 | –0.336593 | H7 | 3.091577 | 2.956558 | –0.918432 | H7 | 4.489788 | 2.549528 | –0.000106 |
| H8 | 2.222961 | –1.788620 | 0.356224 | H8 | 1.905173 | –1.622318 | 0.678988 | H8 | 1.184536 | –1.203739 | 0.000128 |
| H9 | 4.748963 | 1.548627 | –0.517825 | H9 | 4.793589 | 1.145429 | –0.831544 | H9 | 5.220750 | 0.175329 | –0.000104 |
| O10 | 0.385848 | 2.379436 | 0.203975 | O10 | 0.601401 | 2.647497 | –0.193428 | O10 | 1.990182 | 3.295336 | 0.000025 |
| H11 | –0.437072 | 1.853489 | 0.411751 | C11 | –0.158046 | 0.072540 | 0.712690 | C11 | 0.052089 | 1.327661 | 0.000049 |
| C12 | –0.085428 | –0.427188 | 0.626501 | H12 | –0.394746 | –0.935630 | 1.042476 | N12 | –1.001167 | 0.518840 | –0.000093 |
| H13 | –0.141576 | –1.516703 | 0.747734 | N13 | –1.129171 | 0.963045 | 0.723311 | C13 | –2.378738 | 0.980236 | –0.000090 |
| N14 | –1.120789 | 0.312563 | 0.752847 | C14 | –2.502992 | 0.677845 | 1.069897 | H14 | –2.596956 | 1.589028 | 0.882891 |
| C15 | –2.397550 | –0.297162 | 1.046030 | H15 | –2.562112 | 0.163106 | 2.035423 | H15 | –2.597031 | 1.588888 | –0.883185 |
| H16 | –2.309383 | –1.366745 | 1.289900 | H16 | –3.045927 | 1.616163 | 1.175283 | C16 | –3.301633 | –0.228741 | –0.000114 |
| H17 | –2.852761 | 0.198374 | 1.906718 | C17 | –3.206167 | –0.210410 | 0.037444 | O17 | –2.903655 | –1.368959 | –0.000450 |
| C18 | –3.354919 | –0.185019 | –0.139231 | O18 | –2.638511 | –0.918206 | –0.753190 | O18 | –4.577176 | 0.148143 | 0.000195 |
| O19 | –3.024936 | –0.013237 | –1.283320 | O19 | –4.531762 | –0.113333 | 0.180757 | C19 | –5.580310 | –0.915370 | –0.000007 |
| O20 | –4.622206 | –0.346201 | 0.278274 | C20 | –5.349421 | –0.957891 | –0.684258 | H20 | –5.416532 | –1.533786 | 0.884242 |
| C21 | –5.651318 | –0.339205 | –0.749853 | H21 | –5.116989 | –0.706442 | –1.720632 | H21 | –5.416648 | –1.533356 | –0.884577 |
| H22 | –5.597927 | 0.612640 | –1.282062 | H22 | –5.062619 | –1.997514 | –0.514150 | C22 | –6.942063 | –0.256909 | 0.000239 |
| H23 | –5.431054 | –1.136553 | –1.462975 | C23 | –6.799310 | –0.696734 | –0.339453 | H23 | –7.716159 | –1.028462 | 0.000197 |
| C24 | –6.985994 | –0.537239 | –0.063370 | H24 | –7.006732 | –0.944050 | 0.703803 | H24 | –7.079029 | 0.364654 | 0.887471 |
| H25 | –7.016919 | –1.490111 | 0.469487 | H25 | –7.061756 | 0.349670 | –0.508655 | H25 | –7.079216 | 0.364935 | –0.886768 |
| H26 | –7.183912 | 0.266082 | 0.649554 | H26 | –7.439852 | –1.316628 | –0.972015 | H26 | –0.896726 | –0.491412 | –0.000285 |
| H27 | –7.784085 | –0.536441 | –0.810376 | O27 | 4.111252 | –2.489000 | 0.387087 | H27 | –0.156936 | 2.395343 | 0.000200 |
| O28 | 4.533950 | –2.300796 | –0.011787 | O28 | 5.571617 | –1.083152 | –0.394478 | N28 | 3.665606 | –1.999794 | 0.000065 |
| O29 | 5.819657 | –0.608716 | –0.456720 | N29 | 4.437347 | –1.374811 | –0.020476 | O29 | 2.822272 | –2.896191 | 0.000082 |
| N30 | 4.727812 | –1.102087 | –0.193143 | H30 | –0.822244 | 1.891342 | 0.364960 | O30 | 4.879252 | –2.196286 | 0.000088 |
